# Identifying novel data-driven subgroups in congenital heart disease using multi-modal measures of brain structure

**DOI:** 10.1016/j.neuroimage.2024.120721

**Published:** 2024-07-04

**Authors:** Marlee M. Vandewouw, Ami Norris-Brilliant, Anum Rahman, Stephania Assimopoulos, Sarah U. Morton, Azadeh Kushki, Sean Cunningham, Eileen King, Elizabeth Goldmuntz, Thomas A. Miller, Nina H. Thomas, Heather R. Adams, John Cleveland, James F. Cnota, P Ellen Grant, Caren S. Goldberg, Hao Huang, Jennifer S. Li, Patrick McQuillen, George A. Porter, Amy E. Roberts, Mark W. Russell, Christine E. Seidman, Madalina E. Tivarus, Wendy K. Chung, Donald J. Hagler, Jane W. Newburger, Ashok Panigrahy, Jason P Lerch, Bruce D. Gelb, Evdokia Anagnostou

**Affiliations:** a Autism Research Centre, Bloorview Research Institute, Holland Bloorview Kids Rehabilitation Hospital, Toronto, ON, Canada; b Institute of Biomedical Engineering, University of Toronto, Toronto, ON, Canada; c Department of Psychiatry, Icahn School of Medicine, Mount Sinai, New York, USA; d Mouse Imaging Centre, The Hospital for Sick Children, Toronto, ON, Canada; e Translational Medicine, The Hospital for Sick Children, Toronto, ON, Canada; f Department of Medical Biophysics, University of Toronto, Toronto, ON, Canada; g Division of Newborn Medicine, Department of Pediatrics, Boston Children’s Hospital, Boston, MA, USA; h Fetal Neonatal Neuroimaging and Developmental Science Center, Boston Children’s Hospital, Boston, MA, USA; i Department of Pediatrics, Harvard Medical School, Boston, MA, USA; j Department of Genetics, Harvard Medical School, Boston, MA, USA; k Department of Pediatrics, Division of General Pediatrics, University of Utah, Salt Lake City, UT, USA; l Department of Pediatrics, University of Cincinnati, Cincinnati, OH, USA; m Division of Biostatistics and Epidemiology, Cincinnati Children’s Hospital Medical Centre, Cincinnati, OH, USA; n Division of Cardiology, Children’s Hospital of Philadelphia, Philadelphia, PA, USA; o Department of Pediatrics, Perelman School of Medicine, University of Pennsylvania, Philadelphia, PA, USA; p Department of Pediatrics, Maine Medical Center, Portland, ME, USA; q Department of Child and Adolescent Psychiatry and Behavioral Sciences and Center for Human Phenomic Science, The Children’s Hospital of Philadelphia, Philadelphia, PA, USA; r Department of Psychiatry, University of Pennsylvania, Philadelphia, PA, USA; s Departments of Neurology and Pediatrics, University of Rochester Medical Center, Rochester, NY, USA; t Departments of Surgery and Pediatrics, Keck School of Medicine, University of Southern California, LA, USA; u Heart Institute, Cincinnati Children’s Hospital Medical Center, Cincinnati, OH, USA; v Department of Radiology, Boston Children’s Hospital, Boston, MA, USA; w Department of Radiology, Harvard Medical School, Boston, MA, USA; x Department of Pediatrics, C.S. Mott Children’s Hospital, University of Michigan, Ann Arbor, MI, USA; y Department of Radiology, The Children’s Hospital of Philadelphia, Philadelphia, PA, USA; z Department of Radiology, Perelman School of Medicine, University of Pennsylvania, Philadelphia, PA, USA; aa Department of Pediatrics, Duke University Medical Center, Durham, NC, USA; ab Departments of Pediatrics and Neurology, University of California San Francisco, San Francisco, CA, USA; ac Department of Cardiology, Boston Children’s Hospital, Boston, MA USA; ad Division of Genetics and Genomics, Boston Children’s Hospital, Boston, MA, USA; ae Cardiovascular Division, Brigham and Women’s Hospital, Boston, MA, USA; af Howard Hughes Medical Institute, Chevy Chase, MD, USA; ag Department of Imaging Sciences and Department of Neuroscience, University of Rochester Medical Center, Rochester, NY, USA; ah Departments of Pediatrics and Medicine, Columbia University, New York, NY, USA; ai Center for Multimodal Imaging and Genetics, University of California San Diego, USA; aj Department of Radiology, School of Medicine, University of California San Diego, USA; ak Departments of Cognitive Science and Neuroscience, University of California San Diego, USA; al Department of Pediatric Radiology, Children’s Hospital of Pittsburgh, University of Pittsburgh Medical Center, Pittsburgh, PA USA; am Program in Neurosciences & Mental Health, The Hospital for Sick Children, Toronto, ON, Canada; an Wellcome Centre for Integrative Neuroimaging, FMRIB, Nuffield Department of Clinical Neurosciences, University of Oxford, Oxford, UK; ao Mindich Child Health and Development Institute and Department of Pediatrics, Icahn School of Medicine at Mount Sinai, New York, NY USA; ap Institute of Medical Science, University of Toronto, Toronto, ON, Canada

**Keywords:** Congenital heart disease, Brain structure, Genetics, Unsupervised methods, Neurodevelopmental outcomes

## Abstract

Individuals with congenital heart disease (CHD) have an increased risk of neurodevelopmental impairments. Given the hypothesized complexity linking genomics, atypical brain structure, cardiac diagnoses and their management, and neurodevelopmental outcomes, unsupervised methods may provide unique insight into neurodevelopmental variability in CHD. Using data from the Pediatric Cardiac Genomics Consortium Brain and Genes study, we identified data-driven subgroups of individuals with CHD from measures of brain structure. Using structural magnetic resonance imaging (MRI; *N* = 93; cortical thickness, cortical volume, and subcortical volume), we identified subgroups that differed primarily on cardiac anatomic lesion and language ability. In contrast, using diffusion MRI (*N* = 88; white matter connectivity strength), we identified subgroups that were characterized by differences in associations with rare genetic variants and visual-motor function. This work provides insight into the differential impacts of cardiac lesions and genomic variation on brain growth and architecture in patients with CHD, with potentially distinct effects on neurodevelopmental outcomes.

## 1. Introduction

Congenital heart disease (CHD) is the most common congenital anomaly (Hoffman and Kaplan, 2002; Wu et al., 2020), accounting for approximately 3 % of infant deaths (Sadowski, 2009). Significant advances in surgical and medical treatments in the past several decades have greatly reduced mortality for individuals born with CHD (Khairy et al., 2010), making their increased likelihood for neurodevelopmental differences more apparent (Liamlahi and Latal, 2019). Approximately 10 % of children with CHD present with impairments in domains including cognition, motor, language, executive function, social interaction, attention, and impulsivity, increasing to 50 % among individuals with severe forms of CHD (Liamlahi and Latal, 2019; Marino et al., 2012). While patient-specific, postoperative, and preoperative factors have been found to be more predictive of poor neurodevelopmental outcomes in CHD than modifiable intraoperative techniques (Gaynor et al., 2007; Limperopoulos et al., 2010; Miller and McQuillen, 2007; Ortinau et al., 2018), our ability to explain much of the variance remains limited (Gaynor et al., 2007; Goldberg et al., 2019; Marino et al., 2012). Genetic and epigenetic factors have been proposed as possible contributing factors (Limperopoulos et al., 2010), with CHD and neuro-developmental differences potentially sharing genetic etiology (Homsy et al., 2015; Jin et al., 2017).

Neuroimaging is a promising approach to bridge the gap between the diverse neurodevelopmental pathways modulated by CHD-related genetic and non-genetic factors and the variability of neurodevelopmental outcomes in CHD. Magnetic resonance imaging (MRI) studies have shown altered brain maturation *in utero* (Limperopoulos et al., 2010; Ortinau et al., 2018; Rollins et al., 2021), thought to be caused by genetic factors and oxygen and nutrient deficiencies caused by disease-related disruptions in blood flow (Rollins et al., 2021; Sun et al., 2015). This atypical maturation has persistent effects on brain structure, such as reductions in global and regional brain volumes (Bonthrone et al., 2021), predominantly decreased cortical thickness (Verrall et al., 2021; Watson et al., 2017, 2016), reduced white matter integrity (Ehrler et al., 2021; 2020; Rollins et al., 2014; Watson et al., 2018) and altered white matter connectivity (Panigrahy et al., 2015; Schmithorst et al., 2016), which have been shown to be associated with neuro-developmental outcomes (Bonthrone et al., 2021; Ma et al., 2020; Panigrahy et al., 2015; Schmithorst et al., 2016; Verrall et al., 2021; Watson et al., 2018).

Investigations linking the atypical brain structure in CHD and the subsequent behavioral and cognitive sequelae with their genetic underpinnings are an important first step in understanding the complex relationship between genetics, brain, and behavior in CHD. Recent work from the Pediatric Cardiac Genomics Consortium’s (PCGC) Brain and Genes study (ClinicalTrials.gov #NCT01196182) was the first study to compare neurodevelopmental outcomes and brain structure in CHD individuals with (cases) and without (controls) predicted deleterious *de novo* variants (Morton et al., 2022). The study excluded participants with a damaging variant in a gene associated with neurodevelopmental disabilities, designed particularly to identify new genetic sources of variance in neuropsychiatric measures. Although they reported no differences in neurological outcomes between cases and controls, a small group of individuals with rare, loss-of-function (LOF) variants were found to have worse behavioral outcomes and exhibit differences in some measures of brain structure and function(Morton et al., 2022).

Studies investigating CHD have thus far employed classic case-control approaches, where differences in brain structure and/or outcome are compared between pre-specified groups. Given the hypothesized complexity of the relationships linking genomics, the various measures of brain structure, the types of cardiac lesion and their management, and subsequent neurodevelopmental outcomes, unsupervised methods with no *a priori* assumptions may provide unique, complementary insights into the structure of such multivariable data.

In this secondary analysis of the CHD Brain and Genes study, we used clustering techniques on measures of brain structure to identify subgroups of individuals with CHD, beyond case and control definitions. We compared the subgroups on their genetic, clinical, and behavioral characteristics to provide insights into how genomic, cardiac lesion and clinical care features may impact brain structure and ultimately neurodevelopmental outcomes. We performed clustering separately for measures derived from structural MRI (thickness and volume) and diffusion MRI (connectivity strength), allowing for different neural mechanisms and embryological origins to differentially relate to CHD pathology and neurodevelopmental outcomes.

## 2. Materials and methods

### 2.1. Participants

Participants’ data were extracted from the PCGC CHD Brain and Genes study, details of which are outlined in the primary study (Morton et al., 2022). The Pediatric Cardiac Genomics Consortium (PCGC) Brain and Genes study, a cross-sectional study performed from September 2017 to June 2020, included individuals with CHD who were at least eight years of age and who had available research-based genetic sequencing data, and excluded those who had a history of cardiac transplant, underwent a cardiac surgical procedure within six months of enrollment, had a known clinical genetic syndrome, had brain injury that a center investigator determined would overshadow the effect of a genetic variant on neurodevelopmental outcomes, lacked ability to communicate in English or Spanish, or were unable to undergo MRI scanning. Participants were also excluded if they had the presence of a clinically pathogenic copy number variant or damaging coding variant in a gene associated with neurodevelopmental delay; this criterion included both autosomal dominant genes with a single damaging variant or autosomal recessive genes where two variants were present. One hundred participants were recruited for MRI scanning at one of seven participating institutions: Boston Children’s Hospital (BCH; Boston, United States), Children’s Hospital of Philadelphia (CHOP; Philadelphia, United States), the Icahn School of Medicine at Mount Sinai (ISMMS; New York, United States), the University of California, San Francisco (UCSF; San Francisco, United States), the University of Rochester (ROC; Rochester, United States), the University of Utah (UTAH; Salt Lake City, United States), Yale University (YALE; New Haven, United States). Informed consent and assent were obtained from all participants and/or their parents or guardians. A central institutional research ethics board approved the trial’s protocol, and participating sites approved reliance agreements. This secondary analysis was also approved by the Holland Bloorview Kids Rehabilitation Hospital’s research ethics board.

### 2.2. Data acquisition

Information on the nature of the participant’s cardiac lesions and medical histories was obtained from chart review and participant interviews, and included the total number of cardiac surgeries, the total number of open cardiac surgeries, age at first hospital admission, and age of first open cardiac surgery. Participants were classified clinically as having either single ventricle (SV) or biventricular (BV) lesions, and the Fyler coding system was also used to categorize the lesions.

Neuroimaging data were acquired at one of seven participating institutions in the United States. Structural MRI (sMRI) data were obtained with a T1-weighted acquisition and diffusion MRI (dMRI) data were obtained with a multi-shell sequence (see the Supplemental Information and Supplemental Table 1). A neuroradiology reader assessed the structural MRIs for brain injury, providing the number of focal infarcts, the size of focal infarcts if present, and the number of foci with low and high T2 signal; a participant was determined to have MRI findings based on the presence of infarcts and/or foci. Genomic results are described in the primary study (Morton et al., 2022). Participants were classified by heterozygous status for the presence (case) or absence (control) of a damaging *de novo* variant in a gene that was not recognized as a definitive cause of neurodevelopmental disability (NDD; Homsy et al., 2015; Jin et al., 2017) at the time participant stratification (June 2017). Cases were further classified based on whether variants are predicted to cause loss of function (pLOF; Jin et al., 2017; Seidman et al., 2021) in a gene involved in chromatin remodelling (CHR), altered a gene that is highly expressed in the developing brain (HBE), altered a constrained gene with a high probability of intolerance to loss (H-pLI), caused a single abnormal allele in an autosomal recessive NDD gene, or occurred in a gene newly associated with NDD, after participant stratification. Note that this is distinct from the exclusion criteria at study enrollment. The NDD gene list used in this analysis phase does contain NDD genes discovered since enrollment, but most of the individuals with NDD gene variants have a single variant in a NDD gene with an autosomal recessive mechanism (while exclusion criteria required two variants). NDD gene variant status was included in our analysis to fully consider the potential impact of known NDD genes in the cohort, based on updated information at the time of analysis.

Neurocognitive testing was performed in conjunction with, or within six months of the MRI data. For the purposes of this study, we examined primary and secondary measures pre-specified in the original Brain and Genes study (Morton et al., 2022) capturing academic achievement, visual-motor integration, intelligence, memory, executive function and added the Vineland Adaptive Behavior Scales, Third Edition (Vineland-3; Sparrow et al., 2016) from exploratory measures to capture overall adaptive function.

### 2.3. Preprocessing

For the sMRI preprocessing, the Adolescent Brain Cognitive Development (ABCD) Study pipeline was performed (Hagler et al., 2019). The dMRI data were preprocessed and reconstructed using QSIPrep (version 0.14.3; Cieslak et al., 2021), followed by tractography to generate structural connectivity networks of streamline count between pairs of regions in the FreeSurfer cortical and subcortical parcellation. Further details are provided in the Supplemental Information. ComBat harmonization (Fortin et al., 2018) was used to correct for site effects, and the effects of age at imaging (linear and quadratic), sex, and intracranial volume were regressed from each measure. For both steps, bootstrapping (63.2 % of the sample (Efron, 1979), 100,000 iterations) was used to increase robustness (Da-ano et al., 2020). The residual data were *z*-scored for the clustering analysis.

### 2.4. Clustering

The clustering pipeline was run separately for the sMRI and dMRI data. The pipeline consisted of 100,000 bootstrap iterations, each with 63.2 % of the sample, the square root of the number of features (Efron, 1979), and the number of clusters pre-selected as a value between two and five. The difference between pairs of participants was computed for each selected feature, converted to a similarity matrix (Ontanon, 2020), and averaged across all features; spectral clustering (Ng et al., 2001) was then performed using the pre-selected number of clusters. These 100,000 clustering solutions were collected and used to generate a participant co-assignment matrix containing the probability pairs of participants were clustered together across the bootstrap iterations. Finally, hierarchical clustering was performed on the co-assignment matrix. Hierarchical clustering produces a dendrogram, or clustering decision tree, where for each layer (two to five considered in this study) a root node is split into two leaves. Further details on the clustering pipeline are provided in the Supplemental Information.

### 2.5. Statistics

Differences in participant demographics, CHD lesion categories, genetic categories, MRI findings, and behavioural measures between pairs of leaves in each layer of the dendrograms were evaluated using chi-squared tests for categorical variables, ordinal logistic regression for ordinal variables, and either one-way analysis of variances (ANOVAs) or Kruskal-Wallis tests for continuous variables depending on normality; participants with missing data were excluded on a case-by-case basis, and significance was held at *p* < 0.05, uncorrected. Differences in the brain measures between the leaf clusters were also tested and resulting p-values were corrected across the brain regions (Benjamini and Hochberg, 1995), holding significance at *p*_corr_<0.05.

## 3. Results

### 3.1. Participant demographics

**Fig. 1.**
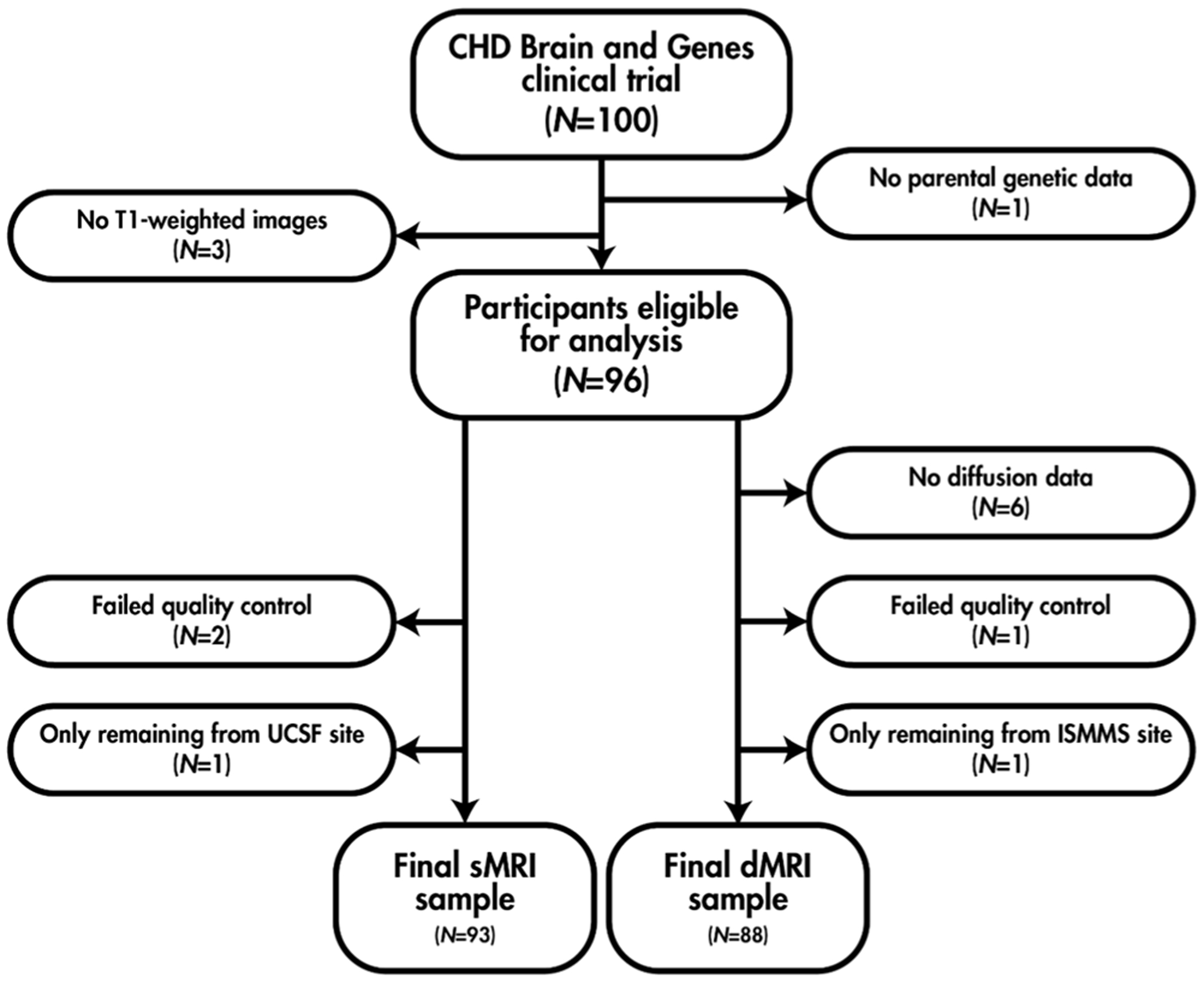
Participant flowchart for the sMRI and dMRI analyses.

Of the 100 participants in the CHD Brain and Genes clinical trial, 93 and 88 were included in the sMRI and dMRI analyses, respectively (see Fig. 1). Sample descriptors are summarized for the sMRI and dMRI datasets in Table 1. To provide consistency with the PCGC CHD Brain and Genes study, sample characteristics summarized by case (damaging *de novo* variant) and control (no such variant) are also presented in Supplemental Tables 2 and 3. MRI findings and descriptive statistics of the behavioral and medical history measures for the sMRI and dMRI datasets are provided in Supplemental Tables 4 and 5, respectively.

### 3.2. sMRI clustering

**Fig. 2.**
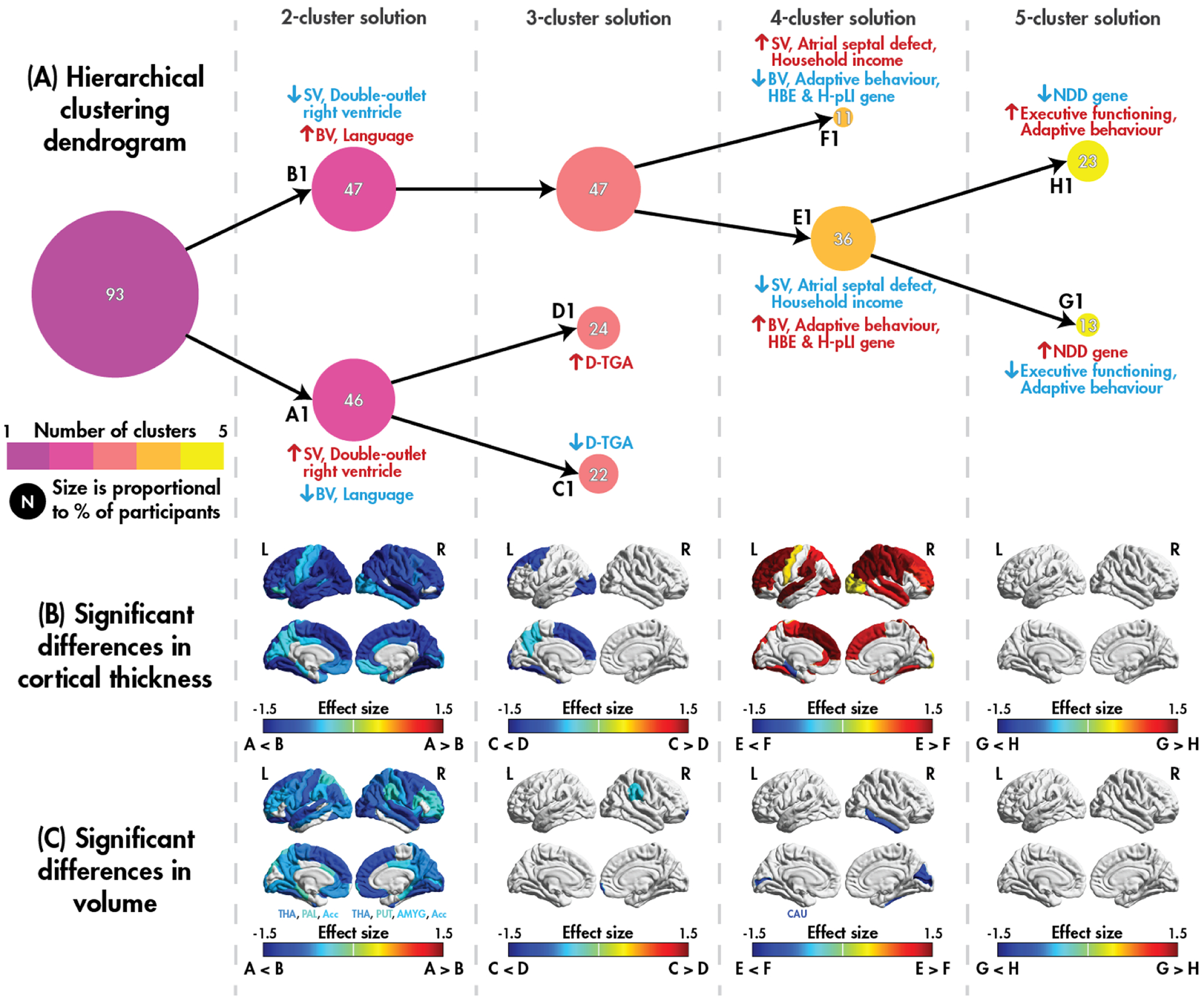
sMRI clustering. A hierarchical clustering dendrogram (A) displaying the emergence clusters for the sMRI dataset; for each dendrogram layer, significant (*p* < 0.05) pairwise differences in the sample characteristics between the leaf clusters (subgroups A1 – H1) are illustrated, with increases indicated by the colour red, and decreases by blue. Effect sizes for significant (*p*_corr_ < 0.05) pairwise differences in cortical thickness (B) and cortical and subcortical volume (C) between the leaf clusters.

Clustering results for the sMRI dataset are presented in Fig. 2. The emergence of the five clusters is presented using the hierarchical clustering dendrogram (Fig. 2A), and the effect sizes of significant differences (*p*_corr_ < 0.05) in cortical thickness (Fig. 2B) and cortical and subcortical volume (Fig. 2C) are also presented. Significant differences (*p* < 0.05) in the participant demographics, CHD lesion categories, genetic categories, and behavioral measures between each pair of leaf clusters are illustrated, with descriptive statistics of significant findings summarized in Table 2; full details are provided in Supplemental Table 6.

In the first layer of the dendrogram, the sample is split into Subgroup-A1 and Subgroup-B1, which show differences in CHD category and language abilities. Specifically, a higher proportion of the individuals with SV lesions were classified into Subgroup-A1 compared to Subgroup-B1, and the SV-enriched subgroup performed worse on all three language measures compared to the BV-dominant subgroup. The SV-enriched subgroup was characterized by widespread decreases in cortical thickness, cortical volume, and subcortical volume, with nearly all regions showing significant differences. As an exploratory analysis, we also performed within-lesion comparisons between the leaf clusters; results are presented in the Supplemental Information.

In the second layer of the dendrogram, the SV-enriched Subgroup-A1 was split into Subgroup-D1 and Subgroup-C1, who differed in the proportion of individuals with a d-transposition of the great arteries: all eight of the participants with this lesion belonged to Subgroup-D1. Localized increases in cortical thickness and cortical volume were observed in Subgroup-D1.

In the next layer, the BV-dominant Subgroup-B1 was split into Subgroup-E1 and Subgroup-F1, which differed in CHD diagnosis, the presence of a pLOF variant in a HBE gene, the presence of a pLOF variant in a H-pLI gene, and the socialization domain of adaptive functioning. Subgroup-F1 contained over half the SV individuals, and Subgroup-E1 contained over 80 % of the BV individuals as well as most of the individuals with a pLOF variant in HBE and H-pLI genes (14 of these individuals had co-occurring variants, eight of whom had a single variant that met criteria for both). However, despite having a decreased genetic impact, the subgroup with most SV individuals had a 14-point reduction in the socialization domain of adaptive functioning compared to Subgroup-E1. Subgroup-F1 had widespread decreased cortical thickness in all lobes, and localized increased volume compared to Subgroup-E1.

Finally, in the last layer of the dendrogram presented, the BV-dominant subgroup with more individuals with pLOF variants (Subgroup-E1) was split into Subgroup-G1 and Subgroup-H1, which differed in the presence of a pLOF variant in an gene newly identified as a dominant neurodevelopmental disability gene or a single variant in a recessive NDD gene (detailed in *Data acquisitions*), in executive functioning and in the communication domain of adaptive functioning. However, the sample size at this layer is small, and no differences in thickness and volume survived multiple comparisons.

### 3.3. dMRI clustering

**Fig. 3.**
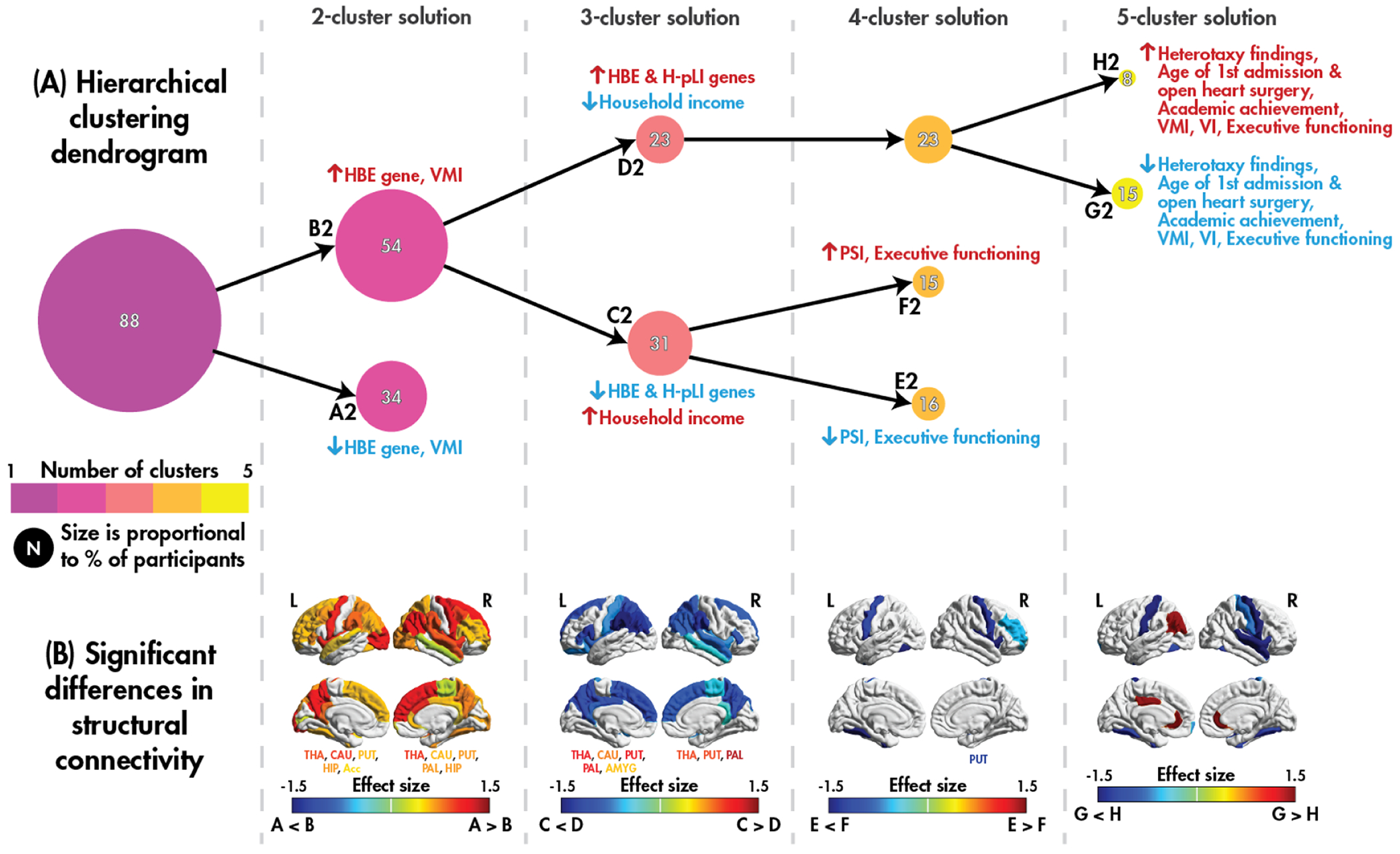
dMRI clustering. A hierarchical clustering dendrogram (A) displaying the emergent clusters for the dMRI dataset; for each dendrogram layer, significant (*p* < 0.05) pairwise differences in the sample characteristics between the leaf clusters (subgroups A2 – H2) are illustrated, with increases indicated by the colour red, and decreases by blue. Effect sizes for significant (*p*_corr_ < 0.05) pairwise differences in cortical thickness (B) and cortical and subcortical volume (C) between the leaf clusters.

Clustering results for the dMRI dataset are presented in Fig. 3, with a summary of significant differences between leaf clusters in the sample characteristics provided in Table 3 (full details provided in Supplemental Table 7).

In the first layer of the dendrogram, the sample is split into Subgroup-A2 and Subgroup-B2, which differ in the presence of a rare pLOF variant in an HBE gene and visual motor integration. Subgroup-B2 contained a higher proportion of those with an HBE gene and had better visual motor integration compared to Subgroup-A2. Subgroup-B2 had decreased structural connectivity strength in regions throughout the brain, spanning all lobes and subcortical structures.

In the next layer of the dendrogram, the HBE-dominant Subgroup-B2 was split into Subgroup-C2 and -D2 who differed in household income level. Given the trend (*p* < 0.07) for the proportion of individuals with a rare pLOF variant in HBE and H-pLI genes, we examined individuals with rare variants in both types of genes (a “combined hit”; 22 individuals, 12 of whom had a single variant that met criteria for both). A significant difference was observed (*X*^2^(54) = 4.13, *p* = 0.04), with Subgroup-D2 having a higher proportion of individuals with a rare HBE and H-pLI variant compared to Subgroup-C2. Subgroup-C2 had decreased structural connectivity strength in cortical regions (primarily in the bilateral parietal and frontal lobes) and increased strength in subcortical regions compared to Subgroup-D2.

In the fourth layer of the dendrogram, Subgroup-C2, the group with fewer individuals with combined HBE and H-pLI genetic hits, was split into Subgroup-E2 and -F2, which differed in processing speed index and executive functioning. Subgroup-F2 performed better on these measures compared to Subgroup-E2 and had localized increases in connectivity strength compared to Subgroup-E2.

In the final layer of the dendrogram, the HBE and H-pLI-dominant subgroup (Subgroup-D2) was split into Subgroup-G2 and -H2, which differed in several clinical and behavioural variables. However, results should be interpreted with the small sample size at this stage of the dendrogram in mind.

## 4. Discussion

Here, we undertook an unsupervised data-driven approach to show that in patients with CHD, specific measures of brain structure can delineate subgroups that have differential associations with cardiac diagnosis and related clinical characteristics, genetics, and ultimately neurodevelopmental outcomes. In general, groups based on cortical thickness and volume are more likely to reflect differences in basic cardiac anatomy and are associated with differences in language performance, whereas groups based on brain structural connectivity are more likely to reflect genomic variation and are mostly associated with differences in visual-motor integration.

Our study uses the first cohort of individuals with CHD for whom extensive genetic and neuroimaging measures had been obtained. While cortical and subcortical morphology and white matter connectivity are known to be influenced by both genetic, environmental, and CHD-specific factors (Bonthrone et al., 2021; Panigrahy et al., 2015; Verrall et al., 2021; Winkler et al., 2010; Yeh et al., 2016), we demonstrate that unsupervised data-driven approaches can identify associations between distinct brain measures and lesion- and genetic-specific influences of CHD on neurodevelopmental outcomes. These observations improve our understanding of the complex and heterogeneous relations between genetics, brain, and behavior in CHD which have not yet been apparent using classic case-control approaches. Our data contributes to the growing body of literature advocating for data-driven approaches to enhance our understanding of differences in brain development and the associated heterogeneity in both the factors contributing to its origins and downstream developmental trajectories of behavior and cognition (Zhang et al., 2020).

When using sMRI measures (thickness and volume), the primary division revealed two subgroups characterized by significantly different proportions of the single-ventricle and biventricular participants, with the subgroup with more single-ventricle individuals being associated with decreased thicknesses and volumes and decreased performance on language measures. The observation confirms the idea that the presence of a single ventricle has profound effect on brain structure which may be independent of a genetic effect and aligns with previous literature reporting widespread decreases in volumes and thickness across the lifespan as well as decreased language abilities in single ventricle cohorts (Brosig et al., 2013; Burns et al., 2021; Claessens et al., 2016; Clouchoux et al., 2013; Hiraiwa et al., 2020; Hövels-Gürich et al., 2008; Mahle et al., 2000; Rajagopalan et al., 2018; Singh et al., 2018; Verrall et al., 2021; Watson et al., 2017). Lower *in utero* substrate delivery to the brain may contribute, in part, to diminished brain volume and cortical thickness, which can increase the likelihood of neurodevelopmental differences. This is supported by the observation that most of the individuals with d-TGA, who are expected to also have poor substrate and oxygen delivery, were clustered into the subgroup that was single-ventricle enriched.

Decreased cortical thickness and cortical volume were observed in the single-compared to bi-ventricular subgroup throughout the brain. Decreased cerebral volume in individuals with CHD compared to typical development has been reported throughout the lifespan, with some evidence of larger reductions in individuals with single-ventricular and cyanotic CHD (see (Bonthrone et al., 2021) for a review), aligning with our findings. For cortical thickness, the largest effects were observed in bilateral entorhinal cortices (part of the medial temporal lobe), pars orbitales (part of the inferior frontal gyrus), and isthmus of the cingulate gyri, and for cortical volume, some of the largest effects were also observed in similar regions (specifically, the right caudal anterior cingulate gyri and the right entorhinal cortex). Decreases in the volumes and thicknesses of frontal, temporal, and cingulate regions have been reported in severe CHD compared to controls throughout development (Cordina et al., 2014; Ortinau et al., 2012; Singh et al., 2018). The pars orbitalis is a well-established region in the language processing network (Price, 2010). Furthermore, the entorhinal cortices and cingulate gyri are part of the limbic system, which is frequently implicated memory (Rolls, 2015), a function that has classically been viewed as distinct from language. However, recent advances in our understanding of cross-function relationships have proposed a language-and-memory network (Banjac et al., 2021b, 2021a; Roger et al., 2022; 2020). This network is thought to be responsible for the intersection of language and declarative memory and includes the entorhinal cortex and isthmus of the cingulate gyri (Roger et al., 2022). Together, this suggests that alterations in the cortical thickness and volume of these brain regions may be the underlying mechanism accompanying differences in language ability. This is supported by evidence in the existing literature, which has shown associations between whole brain and temporal lobe volumes and language ability in CHD (Jakab et al., 2019; Meuwly et al., 2019).

Further division of the biventricular-dominant group revealed two subgroups that contained statistically different proportions of individuals with rare pLOF variants in addition to lesion type. In this higher performing group with predominantly more benign cardiac lesions, the influence of genomic variation starts to become apparent. However, even within this group, those with single ventricles had fewer rare genetic variants and decreased performance, highlighting, again, the impact of single ventricle physiology in the fetal, perinatal, and peri- and postoperative periods on brain development (Clouchoux et al., 2013; Kelly et al., 2017; Newburger et al., 2012; Sun et al., 2015; Zeng et al., 2015).

We chose to investigate structural connectivity separately from brain volume and cortical thickness given their distinct embryological origin, developmental timing, and effects on development. In the first division of the structural connectivity analysis, we identified two subgroups that differed in the proportion of those with rare pLOF variants in HBE genes. The subgroup with more genetic variants was associated with decreased white matter connectivity strength compared to the group with fewer such variants. Despite both groups scoring below average in visual-motor integration, as expected from previous studies (Bellinger and Newburger, 2016; Schaefer et al., 2013; Teixeira et al., 2021), the subgroup with more genetic variants performed better on visuomotor integration. This is the first report of structural connectivity differences between subgroups with differing proportions of pLOF variants. While the primary study examined measures of white matter integrity, finding differences between those with and without a variants in an NDD risk gene, white matter connectivity was not examined (Morton et al., 2022). Some of the largest effect sizes of connectivity strength differences between these two subgroups were observed in the bilateral precentral gyri, right inferior parietal lobule, left lateral occipital cortex and bilateral insulae, amongst others. The primary motor cortex is located within the precentral gyrus (Banker and Tadi, 2021), while the lateral occipital cortex is associated with both the dorsal and ventral visual streams (Goodale and Milner, 2018, 1992), and the parietal lobules and insulae have been shown to be important visuomotor integration (Andersen, 1987; Cauda et al., 2011; Sepulcre, 2014). Together, our results suggest a link between pLOF variants in HBE genes in individuals with CHD with white matter connectivity strength in visuomotor-related regions, which has functional implications. In the subsequent division, the subgroups again separate based on proportion of individuals with pLOF variants, further underscoring that white matter connectivity measures track with genetic influences in CHD.

This study takes a step towards personalized approaches to the care of children with CHD and potential neurodevelopmental concerns. Our current ability to explain the variability of neurodevelopmental conditions in CHD is limited (Gaynor et al., 2007; Goldberg et al., 2019; Marino et al., 2012). This has meant that we are currently unable to confidently determine who is at risk of developing poor outcomes, challenging our ability to deliver anticipatory guidance and secondary prevention interventions before challenges emerge. This calls for a precision medicine approach that takes into consideration each patient’s neurobiology alongside their clinical and genetic factors. As a first step, we identified subgroups of individuals with CHD with specific patterns of brain structure that are associated with less optimal neurodevelopmental outcomes. Although this is not ready for clinical use at this point, as precision medicine advances, subgroups based on brain and other biological markers which predict specific developmental trajectories, will be identified to facilitate the early detection of patients with a high risk of poor neurodevelopmental outcomes. Clinicians then will develop anticipatory guidelines and proactively intervene to optimize outcomes, ultimately improving the quality of life of those affected.

We acknowledge the sample sizes required for reproducible results in neuroimaging studies and the necessity to replicate data-driven approaches in independent samples to enable the clinical translation of findings (Fröhlich et al., 2018; Klapwijk et al., 2021; Marek et al., 2022), and thus the sample size and lack of a replication cohort is a limitation to our study. The sample size increases the risk of type II errors and has also restricted our diffusion features to connectivity strength rather than the raw connectivity values, as otherwise, the number of features would exceed the number of samples, which can lead to less meaningful and interpretable subgroups (Assent, 2012). We also took an exploratory approach to this study given its novelty and our limited sample size, and thus did not correct for multiple comparisons across the behavioural measures, to avoid missing important signal to be explored in future studies. However, we are the first to use such approaches with extensive neuroimaging and genetic measures collected on a cohort of individuals with congenital heart disease and as such no replication cohort is available yet. Our work highlights the importance of understanding both genomic influences and heart anatomy and related factors to influence neurodevelopmental outcomes in individuals with CHD, and advocates for future studies to collect neuroimaging, genetic, clinical, and behavioural measures to allow for further delineation of such influences in large samples and the ability to independently replicate.

This study had several other limitations. We have used cross-sectional data, which only captures neurobiology and cognition at a specific point in time. Future studies should take a longitudinal approach, tracking developmental trajectories of brain structure and connectivity to better understand their impact on neurodevelopmental outcomes in CHD; in particular, the stability of data-driven subgroups across development should be investigated. Other limitations include only studying rare pLOF variants in four gene categories and the exclusion of participants with genetic variants known to be associated with neurodevelopmental delay or impairment. We also did not evaluate for subdural hemorrhage and other known confounding morbidities that affect neurodevelopmental outcomes such as history of prematurity, access to care, race, ethnicity, and the cumulative burden of ionizing radiation from medical imaging procedures (Pasqual et al., 2021). We did consider whether household income and maternal education level as a proxy for social determinants of health contributes to the subgroupings; however, in our dataset we see its contributions further in the dendrogram suggesting that much larger sample sizes may be required to understand the impact of social determinants of health. We also used the FreeSurfer gray matter cortical and subcortical parcellation for diffusion tractography, which may not be the most suitable due to the unreliability of tensor fitting near gray matter regions; however, using the same parcellation for the sMRI and dMRI clustering procedure ensured we were able to make cross-modality comparisons. Of note, we have only used measures of neurobiology to derive the subgroups; while neurobiology acts as an intermediary between the diverse neurodevelopmental pathways modulated by clinical, genetic, and environmental factors and the variability of outcomes in CHD, future work could also use these factors to inform the subgroups.

In conclusion, we used a data-driven approach to investigate the relation between patterns of brain structure/connectivity, rare genomic variation and types of cardiac lesions and behaviour and cognition in individuals with CHD. We identified that whereas measures of cortical thickness and cortical and subcortical volumes mostly relate to cardiac lesion anatomical type and subsequent language outcomes, white matter connectivity strength was more associated with the presence or absence of rare loss-of-function genetic variants and subsequent visuo-motor integration differences. Such insights are foundational to our understanding of distinct impacts of cardiac anatomy versus genomic variation on brain structure and associated neurodevelopmental effects.

## Supplementary Material

Supplementary materials

Supplementary material associated with this article can be found, in the online version, at doi:10.1016/j.neuroimage.2024.120721.

## Figures and Tables

**Table 1:** Sample characteristics for the sMRI and dMRI datasets.

		sMRI	dMRI
N		93	88
Age^1^ range (years)		7 – 41	7 – 41
Median age^1^ (± IQR)		16.1 ± 12.4	16.6 ± 12.5
Sex (M:F)		37:56	38:50
Race (White:Black/African American:Asian:Mixed)		88:2:1:2	83:2:1:2
Ethnicity (Not Hispanic/Latino:Hispanic/Latino)		84:9	80:8
Household income level (Level 1:2:3:4:5:6)^2^		3:7:10:10:16:24	2:6:11:9:15:22
Mother’s education level (Level 1:2:3)^3^		13:53:26	11:51:25
Damaging *de novo* variant present		46	44
Single ventricle (SV)		24	22
Biventricular (BV)		69	66
pLOF variant present	HBE gene	58	55
	CHR gene	8	8
	NDD gene	7	6
	H-pLI gene	36	37

sMRI: structural magnetic resonance imaging; dMRI: diffusion magnetic resonance imaging; IQR: interquartile range; M: male; F: female; CHD: congenital heart disease; BV: biventricular; SV: single-ventricle; pLOF: putative loss-of-function; HBE: high brain expressed gene; CHR: chromatin remodelling gene; NDD: gene newly identified as a dominant neurodevelopmental disability gene or a single variant in a recessive NDD gene; H-pLI: constrained gene with a high probability of intolerance to loss

1 Age at imaging

2 1=<$24,999/year, 2=$25,000-$49,999/year, 3=$50,000-$74,999/year, 4=$75,000-$99,999/year, 5=$100,000-$149,999/year, 6=>$150,000.

3 1=high school or under/other, 2=some college/college, 3=post graduate degree.

**Table 2:** Summary of the significant differences in the sample characteristics for each layer of the sMRI dendrogram.

Dendrogram layer	Measure	Statistics^1^			Subgroup	Descriptive statistics
		Test statistic	*p*-value	Effect size		
2-cluster solution	CHD category (SV/BV)	5.91	0.02	0.25	A1	17 SV, 29 BV
					B1	7 SV, 40 BV
	Oral language composite^2^	7.28	0.01	0.08	A1	103.25 ± 12.75
					B1	110.80 ± 13.71
3-cluster solution	D-TGA	8.88	2.89 × 10^−3^	0.44	C1	0 with, 22 without
					D1	8 with, 16 without
4-cluster solution	CHD category (SV/BV)	5.22	0.02	0.33	E1	33 BV, 3 SV
					F1	7 BV, 4 SV
	Household income level^3^	7.10	0.01	0.19	E1	2:2:6:7:4:6
					F1	1:0:0:0:3:7
	HBE gene	6.65	0.01	0.38	E1	28 with, 8 without
					F1	4 with, 7 without
	H-pLI gene	4.56	0.03	0.31	E1	16 with, 20 without
					F1	1 with, 10 without
	Socialization^4^	17.62	1.41 × 10^−4^	0.30	E1	104.41 ± 8.81
					F1	90.56 ± 8.76
5-cluster solution	NDD gene	5.79	0.02	0.40	G1	3 with, 10 without
					H1	0 with, 23 without
	Category fluency^4^	4.64	0.04	0.12	G1	10.08 ± 2.75
					H1	12.35 ± 3.19
	Communication^4^	5.63	0.02	0.04	G1	96.85 ± 13.07
					H1	105.47 ± 6.50

CHD: congenital heart disease; d-TGA: d-transposition of the great arteries; HBE: high brain expressed gene; H-pLI: constrained gene with a high probability of intolerance to loss; NDD: gene newly identified as a dominant neurodevelopmental disability gene or a single variant in a recessive NDD gene.

1 Normally distributed and continuous: one-way ANOVA *F*-statistic, eta-squared effect size, and mean ± standard deviation, non-normally distributed and continuous: Kruskal-Wallis *H*-statistic, eta-squared effect size, and median [interquartile range], categorical: Pearson chi-squared test statistic, Cramer’s *V* effect size, and sample size per group, ordinal: Ward’s test statistic, pseudo *R*^2^ effect size, and sample size per group.

2 Wechsler Individual Achievement Test’s oral language composite.

3 1=<$24,999/year, 2=$25,000-$49,999/year, 3=$50,000-$74,999/year, 4=$75,000-$99,999/year, 5=$100,000-$149,999/year, 6=>$150,000.

4 Vineland Adaptive Behaviour Scales, Third Edition 4Delis Kaplan Executive Function System.

**Table 3:** Summary of the significant differences in the sample characteristics for each layer of the dMRI dendrogram.

Dendrogram layer	Measure	Statistics^1^			Subgroup	Descriptive statistics
		Test statistic	*p*-value	Effect size		
2-cluster solution	HBE gene	7.99	4.95 × 10^−3^	0.30	A2	15 with, 19 without
					B2	40 with, 14 without
	Visual motor integration^2^	4.26	0.04	0.05	A2	82.29 ± 13.42
					B2	89.00 ± 15.66
3-cluster solution	Household income level^3^	4.25	0.04	0.10	C2	0:1:5:1:9:11
					D2	1:1:4:4:2:3
	HBE and H-pLI gene	4.13	0.04	0.28	C2	9 with, 22 without
					D2	13 with, 10 without
4-cluster solution	Processing speed index^4^	4.82	0.04	0.14	E2	89.31 ± 13.62
					F2	100.67 ± 15.16
	Letter fluency^5^	5.45	0.03	0.16	E2	8.69 ± 2.33
					F2	11.20 ± 3.57
	Category fluency^5^	7.26	0.01	0.20	E2	8.94 ± 2.33
					F2	12.20 ± 4.07
5-cluster solution	Heterotaxy findings	4.11	0.04	0.42	G2	0 with, 15 without
					H2	2 with, 6 without
	Age of 1st admission	4.69	0.03	0.25	G2	2.00 [0.00, 36.00]
					H2	217.00 [45.75, 4458.25]
	Age of 1st open heart surgery	7.09	0.01	0.26	G2	7.50 [3.50, 58.50]
					H2	218.00 [185.50, 4458.25]
	Word reading^6^	12.51	1.95 × 10^−3^	0.37	G2	100.27 ± 14.80
					H2	120.75 ± 9.33
	Sentence comprehension^6^	7.14	0.01	0.25	G2	101.33 ± 14.41
					H2	116.12 ± 8.03
	Math computation^6^	6.54	0.02	0.24	G2	88.73 ± 18.36
					H2	106.12 ± 7.04
	Reading composite^6^	11.60	2.66 × 10^−3^	0.36	G2	100.13 ± 15.27
					H2	119.38 ± 5.73
	Visual motor integration^2^	4.70	0.04	0.18	G2	84.80 ± 16.96
					H2	99.50 ± 11.99
	Verbal comprehension^4^	4.92	0.04	0.19	G2	103.80 ± 14.71
					H2	118.00 ± 14.46
	Category fluency^5^	4.71	0.04	0.18	G2	9.73 ± 3.61
					H2	13.12 ± 3.48
	Category switching^5^	5.22	0.03	0.20	G2	9.13 ± 3.83
					H2	12.50 ± 2.14
	Number sequencing^5^	5.24	0.02	0.21	G2	9.00 [6.50, 11.00]
					H2	11.50 [11.00, 12.50]
	Letter sequencing^5^	8.26	0.01	0.28	G2	8.80 ± 3.19
					H2	12.25 ± 1.49

HBE: high brain expressed gene; H-pLI: constrained gene with a high probability of intolerance to loss.

1 Normally distributed and continuous: one-way ANOVA *F*-statistic, eta-squared effect size, and mean ± standard deviation, non-normally distributed and continuous: Kruskal-Wallis *H*-statistic, eta-squared effect size, and median [interquartile range], categorical: Pearson chi-squared test statistic, Cramer’s *V* effect size, and sample size per group, ordinal: Ward’s test statistic, pseudo *R*^2^ effect size, and sample size per group.

2 Beery-Buktenica Developmental Test of Visual-Motor Integration.

3 1=<$24,999/year, 2=$25,000-$49,999/year, 3=$50,000-$74,999/year, 4=$75,000-$99,999/year, 5=$100,000-$149,999/year, 6=>$150,000.

4 Wechsler intelligence quotient scales.

5 Delis Kaplan Executive Function System.

6 Wide Range Achievement Test, Fourth Edition.

## Data Availability

Data from the Pediatric Cardiac Genomics Consortium Brain and Genes study will be available at the time of publication as per NIH policy.

## References

[R1] Andersen RA, 1987. Inferior parietal lobule function in spatial perception and visuomotor integration. Compr. Physiol 483–518. 10.1002/CPHY.CP010512.

[R2] Assent I, 2012. Clustering high dimensional data. Wiley. Interdiscip. Rev. Data Min. Knowl. Discov 2, 340–350. 10.1002/WIDM.1062.

[R3] Banjac S, Roger E, Cousin E, Perrone-Bertolotti M, Haldin C, Pichat C, Lamalle L, Minotti L, Kahane P, Baciu M, 2021a. Interactive mapping of language and memory with the GE2REC protocol. Brain ImAging Behav. 15, 1562–1579. 10.1007/S11682-020-00355-X/FIGURES/1. 32761343 PMC8286228

[R4] Banjac S, Roger E, Pichat C, Cousin E, Mosca C, Lamalle L, Krainik A, Kahane P, Baciu M, 2021b. Reconfiguration dynamics of a language-and-memory network in healthy participants and patients with temporal lobe epilepsy. Neuroimage Clin. 31, 102702 10.1016/J.NICL.2021.102702. 34090125 PMC8186554

[R5] Banker L, Tadi P, 2021. Neuroanatomy, Precentral Gyrus. StatPearls. 31334938

[R6] Bellinger DC, Newburger JW, 2016. A longitudinal study from infancy to adolescence of the neurodevelopmental phenotype associated with D-transposition of the great arteries. Congenit. Heart Dis. Neurodev 27–40. 10.1016/B978-0-12-801640-4.00003-2. : Understanding and Improving Outcomes.

[R7] Benjamini Y, Hochberg Y, 1995. Controlling the false discovery rate: a practical and powerful approach to multiple testing. J. R. Stat. Soc 57, 289–300.

[R8] Bonthrone AF, Kelly CJ, Ng IHX, Counsell SJ, 2021. MRI studies of brain size and growth in individuals with congenital heart disease. Transl. Pediatr 10, 2171. 10.21037/TP-20-282. 34584889 PMC8429874

[R9] Brosig C, Mussatto K, Hoffman G, Hoffmann RG, Dasgupta M, Tweddell J, Ghanayem N, Mussatto K, 2013. Neurodevelopmental outcomes for children with hypoplastic left heart syndrome at the age of 5 years. Pediatr. Cardiol 34, 1597–1604. 10.1007/s00246-013-0679-3. 23503929 PMC3982227

[R10] Burns J, Varughese R, Ganigara M, Kothare S.v., McPhillips LA, Dhar A, 2021. Neurodevelopmental outcomes in congenital heart disease through the lens of single ventricle patients. Curr. Opin. Pediatr 33, 535–542. 10.1097/MOP.0000000000001052. 34369410

[R11] Cauda F, D’Agata F, Sacco K, Duca S, Geminiani G, Vercelli A, 2011. Functional connectivity of the insula in the resting brain. Neuroimage 55, 8–23. 10.1016/J.NEUROIMAGE.2010.11.049. 21111053

[R12] Cieslak M, Cook PA, He X, Yeh FC, Dhollander T, Adebimpe A, Aguirre GK, Bassett DS, Betzel RF, Bourque J, Cabral LM, Davatzikos C, Detre JA, Earl E, Elliott MA, Fadnavis S, Fair DA, Foran W, Fotiadis P, Garyfallidis E, Giesbrecht B, Gur RC, Gur RE, Kelz MB, Keshavan A, Larsen BS, Luna B, Mackey AP, Milham MP, Oathes DJ, Perrone A, Pines AR, Roalf DR, Richie-Halford A, Rokem A, Sydnor VJ, Tapera TM, Tooley UA, Vettel JM, Yeatman JD, Grafton ST, Satterthwaite TD, 2021. QSIPrep: an integrative platform for preprocessing and reconstructing diffusion MRI data. Nat. Methods 18, 775–778. 10.1038/s41592-021-01185-5, 20217 18. 34155395 PMC8596781

[R13] Claessens NHP, Moeskops P, Buchmann A, Latal B, Knirsch W, Scheer I, Išgum I, de Vries LS, Benders MJNL, von Rhein M, 2016. Delayed cortical gray matter development in neonates with severe congenital heart disease. Pediatr. Res 80, 668–674. 10.1038/pr.2016.145, 20165 80. 27434120

[R14] Clouchoux C, du Plessis AJ, Bouyssi-Kobar M, Tworetzky W, McElhinney DB, Brown DW, Gholipour A, Kudelski D, Warfield SK, McCarter RJ, Robertson RL, Evans AC, Newburger JW, Limperopoulos C, 2013. Delayed cortical development in fetuses with complex congenital heart disease. Cereb. Cortex 23, 2932–2943. 10.1093/CERCOR/BHS281. 22977063

[R15] Cordina R, Grieve S, Barnett M, Lagopoulos J, Malitz N, Celermajer DS, 2014. Brain volumetrics, regional cortical thickness and radiographic findings in adults with cyanotic congenital heart disease. Neuroimage Clin. 4, 319–325. 10.1016/J.NICL.2013.12.011. 24501700 PMC3913831

[R16] Da-ano R, Masson I, Lucia F, Dore M, Robin P, Alfieri J, Rousseau C, Mervoyer A, Reinhold C, Castelli J, De Crevoisier R, Rameé JF, Pradier O, Schick U, Visvikis D, Hatt M, 2020. Performance comparison of modified ComBat for harmonization of radiomic features for multicenter studies. Sci. Rep 10, 1–12. 10.1038/s41598-020-66110-w. 32581221 PMC7314795

[R17] Efron B, 1979. Bootstrap methods: another look at the jackknife. Ann. Stat 7, 1–26.

[R18] Ehrler M, Latal B, Kretschmar O, von Rhein M, O’Gorman Tuura R, 2020. Altered frontal white matter microstructure is associated with working memory impairments in adolescents with congenital heart disease: a diffusion tensor imaging study. Neuroimage Clin. 25, 102123 10.1016/J.NICL.2019.102123. 31869770 PMC6933217

[R19] Ehrler M, Schlosser L, Brugger P, Greutmann M, Oxenius A, Kottke R, O’gorman Tuura R, Latal B, Beatrice L, 2021. Altered white matter microstructure is related to cognition in adults with congenital heart disease. Brain Commun. 3 10.1093/BRAINCOMMS/FCAA224. PMC7811757 33501427

[R20] Fortin JP, Cullen N, Sheline YI, Taylor WD, Aselcioglu I, Cook PA, Adams P, Cooper C, Fava M, McGrath PJ, McInnis M, Phillips ML, Trivedi MH, Weissman MM, Shinohara RT, 2018. Harmonization of cortical thickness measurements across scanners and sites. Neuroimage 167, 104–120. 10.1016/J.NEUROIMAGE.2017.11.024. 29155184 PMC5845848

[R21] Fröhlich H, Balling R, Beerenwinkel N, Kohlbacher O, Kumar S, Lengauer T, Maathuis MH, Moreau Y, Murphy SA, Przytycka TM, Rebhan M, Röst H, Schuppert A, Schwab M, Spang R, Stekhoven D, Sun J, Weber A, Ziemek D, Zupan B, 2018. From hype to reality: data science enabling personalized medicine. BMC Med. 16, 1–15. 10.1186/S12916-018-1122-7/FIGURES/5. PMC6109989 30145981

[R22] Gaynor JW, Wernovsky G, Jarvik GP, Bernbaum J, Gerdes M, Zackai E, Nord AS, Clancy RR, Nicolson SC, Spray TL, 2007. Patient characteristics are important determinants of neurodevelopmental outcome at one year of age after neonatal and infant cardiac surgery. J. Thorac. Cardiovasc. Surg 133, 1344–1353. 10.1016/J.JTCVS.2006.10.087 e3. 17467455 PMC2844117

[R23] Goldberg CS, Hu C, Brosig C, Gaynor JW, Mahle WT, Miller T, Mussatto KA, Sananes R, Uzark K, Trachtenberg F, Pizarro C, Pemberton VL, Lewis AB, Li JS, Jacobs JP, Cnota J, Atz AM, Lai WW, Bellinger D, Newburger JW, 2019. Behavior and quality of life at 6 years for children with hypoplastic left heart syndrome. Pediatrics 144, 20191010. 10.1542/PEDS.2019-1010/76983. PMC6856798 31628208

[R24] Goodale MA, Milner AD, 2018. Two visual pathways – Where have they taken us and where will they lead in future? Cortex 98, 283–292. 10.1016/J.CORTEX.2017.12.002. 29288012

[R25] Goodale MA, Milner AD, 1992. Separate visual pathways for perception and action. Trends Neurosci. 15, 20–25. 10.1016/0166-2236(92)90344-8. 1374953

[R26] Hagler DJ, Hatton SN, Cornejo MD, Makowski C, Fair DA, Dick AS, Sutherland MT, Casey BJ, Barch DM, Harms MP, Watts R, Bjork JM, Garavan HP, Hilmer L, Pung CJ, Sicat CS, Kuperman J, Bartsch H, Xue F, Heitzeg MM, Laird AR, Trinh TT, Gonzalez R, Tapert SF, Riedel MC, Squeglia LM, Hyde LW, Rosenberg MD, Earl EA, Howlett KD, Baker FC, Soules M, Diaz J, de Leon OR, Thompson WK, Neale MC, Herting M, Sowell ER, Alvarez RP, Hawes SW, Sanchez M, Bodurka J, Breslin FJ, Morris AS, Paulus MP, Simmons WK, Polimeni JR, van der Kouwe A, Nencka AS, Gray KM, Pierpaoli C, Matochik JA, Noronha A, Aklin WM, Conway K, Glantz M, Hoffman E, Little R, Lopez M, Pariyadath V, Weiss SR, Wolff-Hughes DL, DelCarmen-Wiggins R, Feldstein Ewing SW, Miranda-Dominguez O, Nagel BJ, Perrone AJ, Sturgeon DT, Goldstone A, Pfefferbaum A, Pohl KM, Prouty D, Uban K, Bookheimer SY, Dapretto M, Galvan A, Bagot K, Giedd J, Infante MA, Jacobus J, Patrick K, Shilling PD, Desikan R, Li Y, Sugrue L, Banich MT, Friedman N, Hewitt JK, Hopfer C, Sakai J, Tanabe J, Cottler LB, Nixon SJ, Chang L, Cloak C, Ernst T, Reeves G, Kennedy DN, Heeringa S, Peltier S, Schulenberg J, Sripada C, Zucker RA, Iacono WG, Luciana M, Calabro FJ, Clark DB, Lewis DA, Luna B, Schirda C, Brima T, Foxe JJ, Freedman EG, Mruzek DW, Mason MJ, Huber R, McGlade E, Prescot A, Renshaw PF, Yurgelun-Todd DA, Allgaier NA, Dumas JA, Ivanova M, Potter A, Florsheim P, Larson C, Lisdahl K, Charness ME, Fuemmeler B, Hettema JM, Maes HH, Steinberg J, Anokhin AP, Glaser P, Heath AC, Madden PA, Baskin-Sommers A, Constable RT, Grant SJ, Dowling GJ, Brown SA, Jernigan TL, Dale AM, 2019. Image processing and analysis methods for the adolescent brain cognitive development study. Neuroimage 202, 116091. 10.1016/J.NEUROIMAGE.2019.116091. 31415884 PMC6981278

[R27] Hiraiwa A, Kawasaki Y, Ibuki K, Hirono K, Matsui M, Yoshimura N, Origasa H, Oishi K, Ichida F, 2020. Brain development of children with single ventricle physiology or transposition of the great arteries: a longitudinal observation study. Semin. Thorac. Cardiovasc. Surg 32, 936–944. 10.1053/J.SEMTCVS.2019.06.013. 31306764

[R28] Hoffman JIE, Kaplan S, 2002. The incidence of congenital heart disease. J. Am. Coll. Cardiol 39, 1890–1900. 10.1016/S0735-1097(02)01886-7. 12084585

[R29] Homsy J, Zaidi S, Shen Y, Ware JS, Samocha KE, Karczewski KJ, DePalma SR, McKean D, Wakimoto H, Gorham J, Jin SC, Deanfield J, Giardini A, Porter GA, Kim R, Bilguvar K, López-Giráldez F, Tikhonova I, Mane S, Romano-Adesman A, Qi H, Vardarajan B, Ma L, Daly M, Roberts AE, Russell MW, Mital S, Newburger JW, Gaynor JW, Breitbart RE, Iossifov I, Ronemus M, Sanders SJ, Kaltman JR, Seidman JG, Brueckner M, Gelb BD, Goldmuntz E, Lifton RP, Seidman CE, Chung WK, 2015. De novo mutations in congenital heart disease with neurodevelopmental and other congenital anomalies. Science 350, 1262–1266. 10.1126/science.aac9396 (1979)1979. 26785492 PMC4890146

[R30] Hövels-Gürich HH, Bauer SB, Schnitker R, Willmes-von Hinckeldey K, Messmer BJ, Seghaye MC, Huber W, 2008. Long-term outcome of speech and language in children after corrective surgery for cyanotic or acyanotic cardiac defects in infancy. Eur. J. Paediatr.Neurol 12, 378–386. 10.1016/J.EJPN.2007.10.004. 18221897

[R31] Jakab A, Meuwly E, Feldmann M, Rhein MV, Kottke R, O’Gorman Tuura R, Latal B, Knirsch W, 2019. Left temporal plane growth predicts language development in newborns with congenital heart disease. Brain 142, 1270–1281. 10.1093/BRAIN/AWZ067. 30957841

[R32] Jin SC, Homsy J, Zaidi S, Lu Q, Morton S, Depalma SR, Zeng X, Qi H, Chang W, Sierant MC, Hung WC, Haider S, Zhang J, Knight J, Bjornson RD, Castaldi C, Tikhonoa IR, Bilguvar K, Mane SM, Sanders SJ, Mital S, Russell MW, Gaynor JW, Deanfield J, Giardini A, Porter GA, Srivastava D, Lo CW, Shen Y, Watkins WS, Yandell M, Yost HJ, Tristani-Firouzi M, Newburger JW, Roberts AE, Kim R, Zhao H, Kaltman JR, Goldmuntz E, Chung WK, Seidman JG, Gelb BD, Seidman CE, Lifton RP, Brueckner M, 2017. Contribution of rare inherited and de novo variants in 2,871 congenital heart disease probands. Nat. Genet 49, 1593–1601. 10.1038/ng.3970. 28991257 PMC5675000

[R33] Kelly CJ, Makropoulos A, Cordero-Grande L, Hutter J, Price A, Hughes E, Murgasova M, Teixeira RPAG, Steinweg JK, Kulkarni S, Rahman L, Zhang H, Alexander DC, Pushparajah K, Rueckert D, Hajnal J.v., Simpson J, Edwards AD, Rutherford MA, Counsell SJ, 2017. Impaired development of the cerebral cortex in infants with congenital heart disease is correlated to reduced cerebral oxygen delivery. Sci. Rep 7 10.1038/S41598-017-14939-Z. PMC5678433 29118365

[R34] Khairy P, Ionescu-Ittu R, MacKie AS, Abrahamowicz M, Pilote L, Marelli AJ, 2010. Changing mortality in congenital heart disease. J. Am. Coll. Cardiol 56, 1149–1157. 10.1016/J.JACC.2010.03.085. 20863956

[R35] Klapwijk ET, van den Bos W, Tamnes CK, Raschle NM, Mills KL, 2021. Opportunities for increased reproducibility and replicability of developmental neuroimaging. Dev. Cogn. Neurosci 47, 100902 10.1016/J.DCN.2020.100902. 33383554 PMC7779745

[R36] Liamlahi R, Latal B, 2019. Neurodevelopmental outcome of children with congenital heart disease. Handb. Clin. Neurol 162, 329–345. 10.1016/B978-0-444-64029-1.00016-3. 31324319

[R37] Limperopoulos C, Tworetzky W, McElhinney DB, Newburger JW, Brown DW, Robertson RL, Guizard N, McGrath E, Geva J, Annese D, Dunbar-Masterson C, Trainor B, Laussen PC, du Plessis AJ, 2010. Brain volume and metabolism in fetuses with congenital heart disease: evaluation with quantitative magnetic resonance imaging and spectroscopy. Circulation 121, 26–33. 10.1161/CIRCULATIONAHA.109.865568. 20026783 PMC2819908

[R38] Ma S, Li Y, Liu Y, Xu C, Li H, Yao Q, Wang Y, Yang Z, Zuo P, Yang M, Mo X, 2020. Changes in cortical thickness are associated with cognitive ability in postoperative school-aged children with tetralogy of fallot. Front. Neurol 11, 691. 10.3389/FNEUR.2020.00691/BIBTEX. 32765405 PMC7380078

[R39] Mahle WT, Clancy RR, Moss EM, Gerdes M, Jobes DR, Wernovsky G, 2000. Neurodevelopmental outcome and lifestyle assessment in school-aged and adolescent children with hypoplastic left heart syndrome. Pediatrics 105, 1082–1089. 10.1542/PEDS.105.5.1082. 10790466

[R40] Marek S, Tervo-Clemmens B, Calabro FJ, Montez DF, Kay BP, Hatoum AS, Donohue MR, Foran W, Miller RL, Hendrickson TJ, Malone SM, Kandala S, Feczko E, Miranda-Dominguez O, Graham AM, Earl EA, Perrone AJ, Cordova M, Doyle O, Moore LA, Conan GM, Uriarte J, Snider K, Lynch BJ, Wilgenbusch JC, Pengo T, Tam A, Chen J, Newbold DJ, Zheng A, Seider NA, Van AN, Metoki A, Chauvin RJ, Laumann TO, Greene DJ, Petersen SE, Garavan H, Thompson WK, Nichols TE, Yeo BTT, Barch DM, Luna B, Fair DA, Dosenbach NUF, 2022. Reproducible brain-wide association studies require thousands of individuals. Nature 603, 654. 10.1038/S41586-022-04492-9. 35296861 PMC8991999

[R41] Marino BS, Lipkin PH, Newburger JW, Peacock G, Gerdes M, Gaynor JW, Mussatto KA, Uzark K, Goldberg CS, Johnson WH, Li J, Smith SE, Bellinger DC, Mahle WT, 2012. Neurodevelopmental outcomes in children with congenital heart disease: evaluation and management a scientific statement from the american heart association. Circulation 126, 1143–1172. 10.1161/CIR.0b013e318265ee8a. 22851541

[R42] Meuwly E, Feldmann M, Knirsch W, von Rhein M, Payette K, Dave H, Tuura ROG, Kottke R, Hagmann C, Latal B, Jakab A, Liamlahi R, Hackenberg A, Kretschmar O, Kellenberger C, Bürki C, Weiss M, 2019. Postoperative brain volumes are associated with one-year neurodevelopmental outcome in children with severe congenital heart disease. Sci. Rep 9, 1–11. 10.1038/s41598-019-47328-9, 20191 9. 31350426 PMC6659678

[R43] Miller SP, McQuillen PS, 2007. Neurology of congenital heart disease: insight from brain imaging. Arch. Dis. Child Fetal. Neonatal. Ed 92, F435. 10.1136/ADC.2006.108845. 17848505 PMC2675385

[R44] Morton SU, Norris-Brilliant A, Cunningham S, King E, Goldmuntz E, Brueckner M, Miller TA, Thomas NH, Liu C, Adams HR, Bellinger DC, Cleveland J, Cnota JF, Dale AM, Frommelt M, Gelbg BD, Grant PE, Goldberg CS, Huang H, Kuperman J, Li JS, McQuillen P, Panigrahy A, Porter GAJ, Roberts AE, Russell M, Seidman CE, Tivarus M, Anagnostou E, Hagler DJJ, Chung WK, Newburger JW, 2022. Association of predicted damaging de novo variants with neurologic outcomes in congenital heart disease: a cross-sectional observational study. JAMA Netw. Open Accepted. 10.1001/jamanetworkopen.2022.53191 PMC9880793 36701153

[R45] Newburger JW, Sleeper LA, Bellinger DC, Goldberg CS, Tabbutt S, Lu M, Mussatto KA, Williams IA, Gustafson KE, Mital S, Pike N, Sood E, Mahle WT, Cooper DS, Dunbar-Masterson C, Krawczeski CD, Lewis A, Menon SC, Pemberton VL, Ravishankar C, Atz TW, Ohye RG, Gaynor JW, 2012. Early developmental outcome in children with hypoplastic left heart syndrome and related anomalies: the single ventricle reconstruction trial. Circulation 125, 2081–2091. 10.1161/CIRCULATIONAHA.111.064113. 22456475 PMC3341507

[R46] Ng A, Jordan M, Weiss Y, Dietterich T, Becker S, Ghahramani Z, 2001. On Spectral Clustering: analysis and an algorithm. Advances in Neural Information Processing Systems. MIT Press.

[R47] Ontañón S, 2020. An overview of distance and similarity functions for structured data. Artif. Intell. Rev 53, 5309–5351. 10.1007/S10462-020-09821-W/FIGURES/2.

[R48] Ortinau C, Beca J, Lambeth J, Ferdman B, Alexopoulos D, Shimony JS, Wallendorf M, Neil J, Inder T, 2012. Regional alterations in cerebral growth exist preoperatively in infants with congenital heart disease. J. Thorac. Cardiovasc. Surg 143 10.1016/J.JTCVS.2011.10.039, 1264–1270.e2. 22143100 PMC3322305

[R49] Ortinau CM, Mangin-Heimos K, Moen J, Alexopoulos D, Inder TE, Gholipour A, Shimony JS, Eghtesady P, Schlaggar BL, Smyser CD, 2018. Prenatal to postnatal trajectory of brain growth in complex congenital heart disease. Neuroimage Clin. 20, 913. 10.1016/J.NICL.2018.09.029. 30308377 PMC6178192

[R50] Panigrahy A, Schmithorst VJ, Wisnowski JL, Watson CG, Bellinger DC, Newburger JW, Rivkin MJ, 2015. Relationship of white matter network topology and cognitive outcome in adolescents with d-transposition of the great arteries. Neuroimage Clin. 7, 438–448. 10.1016/J.NICL.2015.01.013. 25685710 PMC4318874

[R51] Pasqual E, Boussin F, Bazyka D, Nordenskjold A, Yamada M, Ozasa K, Pazzaglia S, Roy L, Thierry-Chef I, de Vathaire F, Benotmane MA, Cardis E, 2021. Cognitive effects of low dose of ionizing radiation – Lessons learned and research gaps from epidemiological and biological studies. Environ. Int 147, 106295 10.1016/J.ENVINT.2020.106295. 33341586

[R52] Price CJ, 2010. The anatomy of language: a review of 100 fMRI studies published in 2009. Ann. N. Y. Acad. Sci 1191, 62–88. 10.1111/J.1749-6632.2010.05444.X. 20392276

[R53] Rajagopalan V, Votava-Smith JK, Zhuang X, Brian J, Marshall L, Panigrahy A, Paquette L, 2018. Fetuses with single ventricle congenital heart disease manifest impairment of regional brain growth. Prenat. Diagn 38, 1042–1048. 10.1002/PD.5374. 30328635

[R54] Roger E, Banjac S, Thiebaut de Schotten M, Baciu M, 2022. Missing links: the functional unification of language and memory (L∪M). Neurosci. Biobehav. Rev 133, 104489 10.1016/J.NEUBIOREV.2021.12.012. 34929226

[R55] Roger E, Pichat C, Torlay L, David O, Renard F, Banjac S, Attyé A, Minotti L, Lamalle L, Kahane P, Baciu M, 2020. Hubs disruption in mesial temporal lobe epilepsy. A resting-state fMRI study on a language-and-memory network. Hum. Brain Mapp 41, 779–796. 10.1002/HBM.24839. 31721361 PMC7268007

[R56] Rollins CK, Ortinau CM, Stopp C, Friedman KG, Tworetzky W, Gagoski B, Velasco-Annis C, Afacan O, Vasung L, Beaute JI, Rofeberg V, Estroff JA, Grant PE, Soul JS, Yang E, Wypij D, Gholipour A, Warfield SK, Newburger JW, 2021. Regional brain growth trajectories in fetuses with congenital heart disease. Ann. Neurol 89, 143–157. 10.1002/ANA.25940. 33084086 PMC7970443

[R57] Rollins CK, Watson CG, Asaro LA, Wypij D, Vajapeyam S, Bellinger DC, Demaso DR, Robertson RL, Newburger JW, Rivkin MJ, 2014. White matter microstructure and cognition in adolescents with congenital heart disease. J. Pediatr 165, 936–944. 10.1016/J.JPEDS.2014.07.028 e2. 25217200 PMC4258111

[R58] Rolls ET, 2015. Limbic systems for emotion and for memory, but no single limbic system. Cortex 62, 119–157. 10.1016/J.CORTEX.2013.12.005. 24439664

[R59] Sadowski SL, 2009. Congenital cardiac disease in the newborn infant: past, present, and future. Crit. Care Nurs. Clin. North Am 21, 37–48. 10.1016/J.CCELL.2008.10.001. 19237042

[R60] Schaefer C, von Rhein M, Knirsch W, Huber R, Natalucci G, Caflisch J, Landolt MA, Latal B, 2013. Neurodevelopmental outcome, psychological adjustment, and quality of life in adolescents with congenital heart disease. Dev. Med. Child Neurol 55, 1143–1149. 10.1111/DMCN.12242. 23937239

[R61] Schmithorst VJ, Panigrahy A, Gaynor JW, Watson CG, Lee V, Bellinger DC, Rivkin MJ, Newburger JW, 2016. Organizational topology of brain and its relationship to ADHD in adolescents with D-transposition of the great arteries. Brain Behav. 6, 504. 10.1002/BRB3.504. PMC4980474 27547505

[R62] Seidman CE, Morton SU, Shimamura A, Newburger PE, Opotowsky AR, Quiat D, Pereira AC, Jin SC, Gurvitz M, Brueckner M, Chung WK, Shen Y, Bernstein D, Gelb BD, Giardini A, Goldmuntz E, Kim RW, Lifton RP, Porter GA, Srivastava D, Tristani-Firouzi M, Newburger JW, Seidman JG, 2021. Association of damaging variants in genes with increased cancer risk among patients with congenital heart disease. JAMa Cardiol. 6, 457–462. 10.1001/JAMACARDIO.2020.4947. 33084842 PMC7578917

[R63] Sepulcre J, 2014. Integration of visual and motor functional streams in the human brain. Neurosci. Lett 567, 68–73. 10.1016/J.NEULET.2014.03.050. 24699175

[R64] Singh S, Kumar R, Roy B, Woo MA, Lewis A, Halnon N, Pike N, 2018. Regional brain gray matter changes in adolescents with single ventricle heart disease. Neurosci. Lett 665, 156. 10.1016/J.NEULET.2017.12.011. 29222023 PMC5801206

[R65] Sparrow SS, Cicchetti D.v., Saulnier CA, 2016. Vineland Adaptive Behavior Scales, 3rd ed. Pearson, Bloomington, MN, United States.

[R66] Sun L, Macgowan CK, Sled JG, Yoo SJ, Manlhiot C, Porayette P, Grosse-Wortmann L, Jaeggi E, McCrindle BW, Kingdom J, Hickey E, Miller S, Seed M, 2015. Reduced fetal cerebral oxygen consumption is associated with smaller brain size in fetuses with congenital heart disease. Circulation 131, 1313–1323. 10.1161/CIRCULATIONAHA.114.013051/-/DC1. 25762062 PMC4398654

[R67] Teixeira J, Caflisch J, Chaouch A, Beck I, Feldmann M, Polentarutti S, Balmer C, Latal B, 2021. Motor and visuomotor function in 10-year-old children with congenital heart disease: association with behaviour. Cardiol. Young 1–6. 10.1017/S1047951121004145. 34643175

[R68] Verrall CE, Yang JYM, Chen J, Schembri A, D’Udekem Y, Zannino D, Kasparian NA, du Plessis K, Grieve SM, Welton T, Barton B, Gentles TL, Celermajer DS, Attard C, Rice K, Ayer J, Mandelstam S, Winlaw DS, Mackay MT, Cordina R, 2021. Neurocognitive dysfunction and smaller brain volumes in adolescents and adults with a fontan circulation. Circulation 143, 878–891. 10.1161/CIRCULATIONAHA.120.048202. 33231097

[R69] Watson CG, Asaro LA, Wypij D, Robertson RL, Newburger JW, Rivkin MJ, 2016. Altered gray matter in adolescents with d-transposition of the great arteries. J. Pediatr 169, 36. 10.1016/J.JPEDS.2015.09.084. 26553098 PMC5854486

[R70] Watson CG, Stopp C, Wypij D, Bellinger DC, Newburger JW, Rivkin MJ, 2018. Altered white matter microstructure correlates with iq and processing speed in children and adolescents post-fontan. J. Pediatr 200, 140–149. 10.1016/J.JPEDS.2018.04.022 e4. 29934026

[R71] Watson CG, Stopp C, Wypij D, Newburger JW, Rivkin MJ, 2017. Reduced cortical volume and thickness and their relationship to medical and operative features in post-Fontan children and adolescents. Pediatr. Res 81, 881–890. 10.1038/pr.2017.30, 20176 81. 28157834

[R72] Winkler AM, Kochunov P, Blangero J, Almasy L, Zilles K, Fox PT, Duggirala R, Glahn DC, 2010. Cortical thickness or grey matter volume? The importance of selecting the phenotype for imaging genetics studies. Neuroimage 53, 1135–1146. 10.1016/J.NEUROIMAGE.2009.12.028. 20006715 PMC2891595

[R73] Wu W, He J, Shao X, 2020. Incidence and mortality trend of congenital heart disease at the global, regional, and national level, 1990–2017. Medicine 99. 10.1097/MD.0000000000020593 (Baltimore). PMC7306355 32502030

[R74] Yeh FC, Vettel JM, Singh A, Poczos B, Grafton ST, Erickson KI, Tseng WYI, Verstynen TD, 2016. Quantifying differences and similarities in whole-brain white matter architecture using local connectome fingerprints. PLoS Comput. Biol 12, e1005203 10.1371/JOURNAL.PCBI.1005203. 27846212 PMC5112901

[R75] Zeng S, Zhou QC, Zhou JW, Li M, Long C, Peng QH, 2015. Volume of intracranial structures on three-dimensional ultrasound in fetuses with congenital heart disease. Ultrasound. Obstet. Gynecol 46, 174–181. 10.1002/UOG.14677. 25270670

[R76] Zhang X, Braun U, Tost H, Bassett DS, 2020. Data-driven approaches to neuroimaging analysis to enhance psychiatric diagnosis and therapy. Biol. Psychiatry Cogn. Neurosci. Neuroimaging 5, 780–790. 10.1016/J.BPSC.2019.12.015. 32127291

