# Supplementary material for: Identifying novel data-driven subgroups in congenital heart disease using multi-modal measures of brain structure
Source: Neuroimage. Author manuscript; Available in PMC 2026 Mar 30. (PMC13034073; doi:10.1016/j.neuroimage.2024.120721)
Supplement: Supplementary materials [file NIHMS2145483-supplement-Supplementary_materials.docx]

**Identifying novel data-driven subgroups in congenital heart disease using multi-modal measures of brain structure**

**Supplemental Information**

**Methods**

***Image acquisition***

Neuroimaging data were acquired across seven institutions with either a Siemens Prisma (BCH, CHOP), Siemens Prisma Fit (UTAH, YALE, ROC), or General Electric (GE) Discovery (ISMMS, UCSF) scanner. Structural MRI (sMRI) data were obtained with a T1-weighted acquisition with an inversion-prepared RF-spoiled gradient echo sequence and diffusion MRI (dMRI) data were obtained with a multi-shell multi-band echo-planar imaging sequence with a slice acceleration factor of 3 ([7, 6, 15, 15, 60] directions of b = [0, 500, 1000, 2000, 3000] s/mm^2^). Acquisition details for the Siemens and GE scanners are summarized in **Supplemental Table 1**. Two field maps with opposing phase-encoding directions were also acquired to correct for image distortions in the dMRI data due to magnetic field inhomogeneities.

***Preprocessing***

For the sMRI preprocessing, the Adolescent Brain Cognitive Development (ABCD) Study pipeline was performed (Hagler et al., 2019). The images were corrected for gradient nonlinearity distortions (Jovicich et al., 2006; Wald et al., 2001) and intensity inhomogeneities (Hagler et al., 2019), and cortical reconstruction and volumetric segmentation was performed with FreeSurfer (version 5.3; Fischl, 2012)). Quality control of the reconstruction was performed by trained technicians who either recommended each image for use or for exclusion (Hagler et al., 2019). Regional morphometric measures (cortical thickness and cortical and subcortical volume) were extracted using the standard FreeSurfer parcellation scheme which consists of 68 cortical and 14 subcortical regions (Desikan et al., 2006; Fischl et al., 2002).

The dMRI data were preprocessed and reconstructed using QSIPrep (version 0.14.3; Cieslak et al., 2021). The T1-weighted image was first bias field corrected (Tustison et al., 2010), skull-stripped using Advanced Normalization Tools (ANTs), segmented into gray matter, white matter, and CSF using FMRIB’s Automated Segmentation Tool (Zhang et al., 2001), and spatially normalized with the ICBM152 nonlinear asymmetrical standard space template (Avants et al., 2008; Fonov et al., 2009). Next, the dMRI data were denoised (Veraart et al., 2016) and corrected for Gibb’s ringing (Kellner et al., 2016), bias field inhomogeneities (Tustison et al., 2010), head motion (Cieslak et al., 2021; Merlet and Deriche, 2013), and susceptibility distortions (Cox and Hyde, 1997); the estimated susceptibility distortion was used to generate an unwarped *b*=0 reference which was used for co-registration with the T1-weighted image using ANTs. Multi-tissue response functions were estimated (Dhollander et al., 2019, 2016) and used to estimate normalized fiber orientation distributions (FODs) using constrained spherical deconvolution (Raffelt et al., 2017; Tournier et al., 2008, 2004). Whole-brain anatomically constrained probabilistic tractography was performed using the second-order integration over FODs (iFOD2) algorithm (Smith et al., 2012; Tournier et al., 2010). Ten million streamlines were generated under the default parameters and were subsequently weighted using spherical-deconvolution informed filtering of tractograms to increase biological plausibility (Smith et al., 2015). Structural connectivity matrices were generated using the FreeSurfer cortical and subcortical parcellation with edge weights defined as the streamline count between pairs of parcels weighted by the inverse of the parcel volumes, and connectivity strength (sum of each parcels connection weights) was computed for each region.

***Clustering analysis***

The clustering pipeline consisted for 100,000 bootstrap iterations. For each bootstrap iteration, 63.2% of the sample was selected resulting in 59 and 56 participants for the sMRI and dMRI analysis, respectively, and the square root of the number of features were selected resulting in 12 and 9 features for the sMRI and dMRI analysis, respectively. The Euclidean distance was used to compute the difference between pairs of participants separately for each feature, and the inverse was taken to convert to a similarity. The arithmetic mean was used to average the similarity matrices across the selected features. Spectral clustering was applied to the averaged similarity matrix, using a pre-specified number of clusters and the k-means method to cluster the eigenvectors of the Laplacian matrix. The 100,000 spectral clustering solutions were collected and used to generate a participant co-assignment matrix containing the probability pairs of participants were clustered together across the bootstrap iterations. The probabilities in the final co-assignment matrix were inverted (e.g., *p*_inv_=1-*p*), and a hierarchical clustering tree was computed using the Ward linkage method.

The Calinski-Harabasz (Calinski and Harabasz, 1974) indices evaluating cluster performance for each layer of the sMRI and dMRI dendrograms are presented in **Supplemental Figure 1**. The optimal number of clusters is determined by maximizing the index, suggesting that two clusters is optimal for both datasets. However, the “elbow rule” (e.g. (Chikumbo and Granville, 2019)) alternatively suggests to select a sharp elbow (inflection point) peak of the curve that is the onset of diminishing returns. Given that the curves are approximately linear in both datasets, the elbow rule suggests there is no reason to prefer one clustering solution over another, and, thus, we examined the full spectrum of solutions.

***sMRI exploratory analysis***

We explored the characteristics of individuals with SV lesions who were classified into the SV-enriched Subgroup-A1 as compared with the SV individuals who belonged to the BV-dominant Subgroup-B1. We found that individuals with SV lesions who were in Subgroup-B1 had their first open heart surgery earlier in life (*W*(1,19) = 3.93, *p* = 0.047), with 80% of individuals having neonatal open heart surgery (≤30 days; *Median* = 3 days, *IQR* =46 days), compared to those in the SV-enriched Subgroup-A1, where only 44% of individuals had their surgery within this time frame (*Median* = 80 days, *IQR* = 197 days). Performing a similar analysis with the BV individuals, a trend in the opposite direction was observed: those with BV lesions were more likely to end up in the BV-dominant Subgroup-B1 (*Median* = 322 days, *IQR* = 2,334 days) compared to the SV-enriched Subgroup-A1 (*Median* = 11 days, *IQR* = 181 days) if they had their first open heart surgery later in life (*W*(1,54) = 3.48, *p* = 0.06). This may suggest that early correction may be associated with better language outcomes in some SV individuals, or that late correction potentially indexes milder conditions in some BV individuals, although directionality cannot be determined by our data.


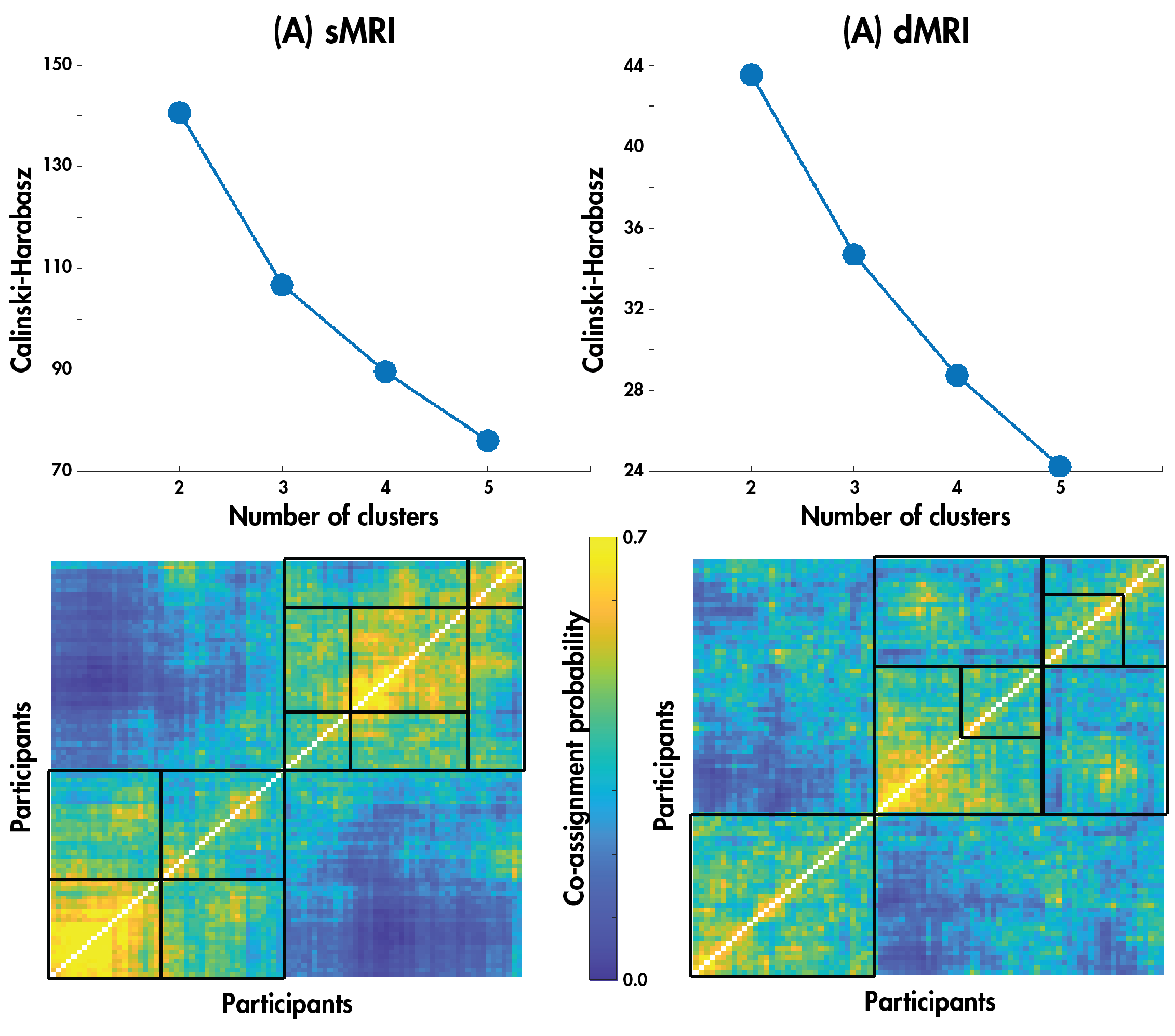


**Supplemental Figure 1**: Calinski-Harabasz indices for each layer in the sMRI (A) and dMRI (B) dendrograms (top) along with the participant co-assignment matrices (bottom), where each cluster is indicated by the black lines.

**Supplemental Table 1**: MRI acquisition protocols for the T1-weighted and diffusion data acquired on the Siemens and GE scanners.

| **Scan Type** | **Scanner** | **TR**  **(ms)** | **TE**  **(ms)** | **FA**  **(°)** | **FOV**  **(mm)** | **Voxel size (mm)** | **Scan time (min)** |
| --- | --- | --- | --- | --- | --- | --- | --- |
| T1-weighted | Siemens | 2500 | 2.88 | 8.0 | 256×256×176 | 1.0 | 7:12 |
|  | GE | 2500 | 2.00 | 8.0 | 256×256×208 | 1.0 | 6:09 |
| Diffusion | Siemens | 4100 | 88.00 | 90.0 | 240×240×138 | 1.7 | 7:31 |
|  | GE | 4100 | 81.90 | 90.0 | 240×240×138 | 1.7 | 7:30 |

GE: General Electric; TR: repetition time; TE: echo time; FA: flip angle; FOV: field of view

**Supplemental Table 2**: Sample characteristics for the sMRI dataset, summarized by case (damaging *de novo* variant) and control (no such variant). Significant differences (*p*<0.05) between cases and controls are bolded.

|  | | **Control** | **Case** | **Statistics** |
| --- | --- | --- | --- | --- |
| **Demographics** | **N** | 47 | 46 | – |
|  | **Age range (years)** | 8 – 39 | 7 – 41 | – |
|  | **Median age (± IQR)** | 16.28 ± 12.71 | 15.11 ± 11.24 | *H*(1,91)=0.02^,^ *p*=0.90 |
|  | **Sex (M:F)** | 15:32 | 22:24 | *X*^2^(93)=2.46^,^ *p*=0.12 |
|  | **Race**  **(White:Black/African American:Asian:Mixed)** | 44:1:1:1 | 44:1:0:1 | *X*^2^(93)=0.99^,^ *p*=0.80 |
|  | **Ethnicity**  **(Not Hispanic/Latino:Hispanic/Latino)** | 43:4 | 41:5 | *X*^2^(93)=0.15^,^ *p*=0.70 |
|  | **Household income level**  **(Level 1:2:3:4:5:6)^1^** | 1:3:4:7:8:13 | 2:4:6:3:8:11 | *W*(1)=0.44, *p*=0.51 |
|  | **Mother’s education level**  **(Level 1:2:3)^2^** | 6:29:12 | 7:24:14 | *W*(1)=0.07, *p*=0.79 |
| **CHD lesion present** | **CHD category (BV:SV)** | 34:13 | 35:11 | *X*^2^(93)=0.17^,^ *p*=0.68 |
|  | **Tetralogy of Fallot** | 5 | 10 | *X*^2^(93)=2.12^,^ *p*=0.15 |
|  | **Double outlet right ventricle** | 7 | 3 | *X*^2^(93)=1.70^,^ *p*=0.19 |
|  | **Atrial septal defect- Secundum** | 17 | 9 | *X*^2^(93)=3.18^,^ *p*=0.07 |
|  | **Hypoplastic left heart syndrome** | 1 | 4 | *X*^2^(93)=1.97^,^ *p*=0.16 |
|  | **Coarctation of the aorta** | 4 | 3 | *X*^2^(93)=0.13^,^ *p*=0.72 |
|  | **D-transposition of the great arteries** | 10 | 3 | ***X*^2^(93)=4.21^,^ *p*=0.04** |
|  | **Isolated ventricular septal defect** | 13 | 14 | *X*^2^(93)=0.09^,^ *p*=0.77 |
|  | **Atrioventricular canal** | 5 | 2 | *X*^2^(93)=1.32^,^ *p*=0.25 |
|  | **Heterotaxy** | 8 | 5 | *X*^2^(93)=0.73^,^ *p*=0.39 |
| **LOF Variant** | **HBE** | 24 | 34 | ***X*^2^(93)=5.17^,^ *p*=0.02** |
|  | **CHR** | 5 | 3 | *X*^2^(93)=0.50^,^ *p*=0.48 |
|  | **NDD** | 3 | 4 | *X*^2^(93)=0.18^,^ *p*=0.67 |
|  | **H-pLI** | 13 | 23 | ***X*^2^(93)=4.89^,^ *p*=0.03** |

sMRI: structural magnetic resonance imaging; IQR: interquartile range; M: male; F: female; CHD: congenital heart disease; BV: biventricular; SV: single-ventricle; LOF: loss-of-function; HBE: high brain expressed gene; CHR: chromatin remodelling gene; NDD: newly identified as a dominant neurodevelopmental disability gene or a single variant in a recessive NDD gene; H-pLI: constrained gene with a high probability of intolerance to loss; ^1^1=<$24,999, 2=$25,000-$49,999, 3=$50,000-$74,999, 4=$75,000-$99,999, 5=$100,000-$149,999, 6=>$150,000; ^2^1=high school or under/other, 2=some college/college, 3=post graduate degree

**Supplemental Table 3**: Sample characteristics for the dMRI dataset, summarized by case (damaging *de novo* variant) and control (no such variant). Significant differences (*p*<0.05) between cases and controls are bolded.

|  | | **Control** | **Case** | **Statistics** |
| --- | --- | --- | --- | --- |
| **Demographics** | **N** | 44 | 44 | – |
|  | **Age range (years)** | 8 – 39 | 7 – 41 | – |
|  | **Median age (± IQR)** | 17.67 ± 12.89 | 14.44 ± 11.44 | *H*(1,86)=0.78, *p*=0.38 |
|  | **Sex (M:F)** | 16:28 | 22:22 | *X*^2^(88)=1.67, *p*=0.20 |
|  | **Race**  **(White:Black/African American:Asian:Mixed)** | 41:1:1:1 | 42:1:0:1 | *X*^2^(88)=1.01^,^ *p*=0.80 |
|  | **Ethnicity**  **(Not Hispanic/Latino:Hispanic/Latino)** | 41:3 | 39:5 | *X*^2^(88)=0.55^,^ *p*=0.46 |
|  | **Household income level**  **(Level 1:2:3:4:5:6)^1^** | 0:3:5:6:8:11 | 2:3:6:3:7:11 | *W*(1)=0.16, *p*=0.69 |
|  | **Mother’s education level**  **(Level 1:2:3)^2^** | 5:28:11 | 6:23:14 | *W*(1)=0.19, *p*=0.66 |
| **CHD lesion present** | **CHD category (BV:SV)** | 32:12 | 34:10 | *X*^2^(88)=0.24, *p*=0.62 |
|  | **Teralogy of Fallot** | 4 | 9 | *X*^2^(88)=2.26, *p*=0.13 |
|  | **Double outlet right ventricle** | 7 | 3 | *X*^2^(88)=1.81, *p*=0.18 |
|  | **Atrial septal defect- Secundum** | 16 | 8 | *X*^2^(88)=3.67, *p*=0.06 |
|  | **Hypoplastic left heart syndrome** | 1 | 4 | *X*^2^(88)=1.91, *p*=0.17 |
|  | **Coarctation of the aorta** | 3 | 3 | *X*^2^(88)=0.00, *p*=1.00 |
|  | **D-transposition of the great arteries** | 10 | 3 | ***X*^2^(88)=4.42, *p*=0.04** |
|  | **Isolated ventricular septal defect** | 13 | 14 | *X*^2^(88)=0.05, *p*=0.82 |
|  | **Atrioventricular canal** | 5 | 2 | *X*^2^(88)=1.40, *p*=0.24 |
|  | **Heterotaxy** | 8 | 5 | *X*^2^(88)=0.81, *p*=0.37 |
| **LOF Variant** | **HBE** | 22 | 33 | ***X*^2^(88)=5.87, *p*=0.02** |
|  | **CHR** | 5 | 3 | *X*^2^(88)=0.55, *p*=0.46 |
|  | **NDD** | 2 | 4 | *X*^2^(88)=0.72, *p*=0.40 |
|  | **H-pLI** | 14 | 23 | *X*^2^(88)=3.78, *p*=0.05 |

sMRI: structural magnetic resonance imaging; IQR: interquartile range; M: male; F: female; CHD: congenital heart disease; BV: biventricular; SV: single-ventricle; LOF: loss-of-function; HBE: high brain expressed gene; CHR: chromatin remodelling gene; NDD: newly identified as a dominant neurodevelopmental disability gene or a single variant in a recessive NDD gene; H-pLI: constrained gene with a high probability of intolerance to loss; ^1^1=<$24,999, 2=$25,000-$49,999, 3=$50,000-$74,999, 4=$75,000-$99,999, 5=$100,000-$149,999, 6=>$150,000; ^2^1=high school or under/other, 2=some college/college, 3=post graduate degree

**Supplemental Table 4**: MRI findings for the sMRI and dMRI datasets.

|  | | **sMRI (*N*=93)** | **dMRI (*N*=88)** |
| --- | --- | --- | --- |
| **MRI findings available** | | 92 | 87 |
| **Focal infarct, number** | **0** | 82 | 77 |
|  | **1-5** | 10 | 10 |
| **Focal infarct, size**  **(if number > 0)** | **<1cm** | 6 | 6 |
|  | **1-3cm** | 3 | 3 |
|  | **>3cm** | 1 | 1 |
| **Foci of low T2, number** | **0** | 91 | 86 |
|  | **1-5** | 1 | 1 |
| **Foci of high T2, number** | **0** | 67 | 62 |
|  | **1-5** | 21 | 20 |
|  | **6-10** | 4 | 4 |
|  | **>20** | 0 | 1 |
| **Any finding (infarct or foci)** | | 33 | 33 |

MRI: magnetic resonance imaging; T2: T2-weighted MRI

**Supplemental Table 5**: Descriptive statistics for the behavioural and medical history measures for the sMRI and dMRI datasets.

|  | | **sMRI** | **dMRI** |
| --- | --- | --- | --- |
| **Medical history** | **# surgeries** | *Median*=2.00, *IQR*=2.00 | *Median*=2.00, *IQR*=2.00 |
|  | **# open heart surgeries** | *Median*=2.00, *IQR*=2.00 | *Median*=2.00, *IQR*=2.00 |
|  | **Age of 1^st^ hospital admission (days)** | *Median*=7.00, *IQR*=416.50 | *Median*=9.00, *IQR*=385.00 |
|  | **Age of 1^st^ open heart surgery (days)** | *Median*=43.00, *IQR*=723.25 | *Median*=45.50, *IQR*=689.00 |
| **WRAT-4** | **Word reading** | *Mean*=106.82, *SD*=13.05 | *Mean*=106.64, *SD*=12.91 |
|  | **Sentence comprehension** | *Mean*=105.94, *SD*=12.58 | *Mean*=106.01, *SD*=12.43 |
|  | **Spelling** | *Mean*=104.26, *SD*=13.84 | *Mean*=103.88, *SD*=13.70 |
|  | **Math computation** | *Mean*=101.09, *SD*=15.90 | *Mean*=101.19, *SD*=16.02 |
|  | **Reading composite** | *Mean*=106.23, *SD*=12.59 | *Mean*=106.17, *SD*=12.36 |
| **Beery-VMI** | **VMI** | *Mean*=86.69, *SD*=14.95 | *Mean*=86.41, *SD*=15.11 |
| **W-IQ** | **Verbal comprehension index** | *Mean*=107.42, *SD*=15.97 | *Mean*=108.01, *SD*=15.59 |
|  | **Perceptual/fluid reasoning index** | *Mean*=100.63, *SD*=13.79 | *Mean*=100.59, *SD*=13.80 |
|  | **Processing speed index** | *Mean*=97.10, *SD*=13.39 | *Mean*=97.23, *SD*=13.74 |
|  | **Full-scale intelligence quotient** | *Mean*=101.62, *SD*=14.46 | *Mean*=101.81, *SD*=14.31 |
| **WRAML-2** | **Immediate recall (story memory)** | *Median*=11.00, *IQR*=3.00 | *Median*=11.00, *IQR*=3.00 |
|  | **Immediate recall (picture memory)** | *Mean*=8.76, *SD*=2.82 | *Mean*=8.84, *SD*=2.82 |
|  | **Delayed recall (picture memory)** | *Median*=10.00, *IQR*=4.00 | *Median*=10.00, *IQR*=4.00 |
|  | **Delayed recall (story memory)** | *Median*=11.00, *IQR*=3.00 | *Median*=11.00, *IQR*=3.00 |
|  | **Delayed recall (story recognition)** | ^1^*Median*=11.00, *IQR*=3.00 | ^5^*Median*=11.00, *IQR*=3.50 |
| **D-KEFS** | **Letter fluency** | *Median*=10.00, *IQR*=5.00 | *Median*=10.00, *IQR*=5.00 |
|  | **Category fluency** | *Median*=11.00, *IQR*=6.00 | *Median*=11.00, *IQR*=6.00 |
|  | **Category switching accuracy** | *Median*=11.00, *IQR*=4.00 | *Median*=11.00, *IQR*=4.00 |
|  | **Visual scanning** | *Median*=10.00, *IQR*=4.00 | *Median*=10.00, *IQR*=4.00 |
|  | **Number sequencing** | *Median*=11.00, *IQR*=3.25 | *Median*=11.00, *IQR*=3.00 |
|  | **Letter sequencing** | *Median*=11.00, *IQR*=4.00 | *Median*=11.00, *IQR*=3.50 |
|  | **Number-letter switching** | *Median*=10.00, *IQR*=5.00 | *Median*=10.00, *IQR*=4.50 |
|  | **Tower total achievement** | *Median*=10.00, *IQR*=4.00 | *Median*=10.00, *IQR*=4.00 |
| **WIAT-III** | **Oral language composite** | ^2^*Mean*=107.06, *SD*=13.71 | ^6^*Mean*=107.17, *SD*=13.47 |
| **Vineland-3** | **Socialization** | ^3^*Mean*=102.62, *SD*=10.62 | ^7^*Mean*=102.92, *SD*=10.35 |
|  | **Communication** | ^3^*Median*=105.00, *IQR*=13.00 | ^8^*Median*=105.00, *IQR*=13.00 |
|  | **Daily living skills** | ^4^*Mean*=103.09, *SD*=13.52 | ^9^*Mean*=103.16, *SD*=13.29 |
|  | **Adaptive behaviour composite** | ^4^*Median*=102.00, *IQR*=17.50 | ^9^*Median*=103.00, *IQR*=18.00 |

sMRI: structural magnetic resonance imaging; dMRI: diffusion magnetic resonance imaging; IQR: interquartile range; WRAT-4: Wide Range Achievement Test, Fourth Edition; Beery-Buktenica Developmental Test of Visual-Motor Integration, Sixth Edition; W-IQ: Wechsler intelligence quotient scales; WRAML-2: Wide Range Assessment of Memory and Learning, Second Edition; D-KEFS: Delis Kaplan Executive Function System; WIAT-III: Wechsler Individual Achievement Test’s oral language composite; Vineland-3: Vineland Adaptive Behavior Scales, Third Edition; ^1^Data available for 77 of the 93 participants; ^2^Data available for 88 of the 93 participants; ^3^Data available for 82 of the 93 participants; ^4^Data available for 81 of the 93 participants; ^5^Data available for 72 of the 88 participants; ^6^Data available for 84 of the 88 participants; ^7^Data available for 78 of the 88 participants; ^8^Data available for 77 of the 88 participants; ^9^Data available for 76 of the 88 participants

**Supplemental Table 6**: Statistical details examining differences in the sample characteristics, behavioural measures, and medical history measures between each pair of leaves in the sMRI dendrogram. For the continuous measures, one-way ANOVAs (indicated by the *F*-statistic) or Kruskal-Wallis (indicated by the *H*-statistic) were performed depending on normality. Significant (*p*<0.05) effects are bolded.

|  |  | **2-cluster** | **3-cluster** | **4-cluster** | **5-cluster** |
| --- | --- | --- | --- | --- | --- |
| **Demo-**  **graphics** | **Age** | *H*=1.04, *p*=0.31 | *H*=2.65, *p*=0.10 | *H*=0.25, *p*=0.62 | *H*=0.46, *p*=0.50 |
|  | **Sex** | *X*^2^=0.02, *p*=0.90 | *X*^2^=0.06, *p*=0.81 | *X*^2^=0.15, *p*=0.70 | *X*^2^=0.45, *p*=0.50 |
|  | **Household income level^1^** | *W*=0.11, *p*=0.74 | *W*=0.36, *p*=0.55 | ***W*=7.10, *p*=0.01** | *W*=0.18, *p*=0.67 |
|  | **Mother’s education level^2^** | *W*=0.02, *p*=0.88 | *W*=0.20, *p*=0.65 | *W*=0.07, *p*=0.79 | *W*=0.44, *p*=0.51 |
|  | **Damaging *de novo* variant present** | *X*^2^=0.87, *p*=0.35 | *X*^2^=0.38, *p*=0.54 | *X*^2^=1.76, *p*=0.18 | *X*^2^=1.08, *p*=0.30 |
|  | **MRI Site** | *X*^2^=8.89, *p*=0.11 | *X*^2^=3.09, *p*=0.54 | *X*^2^=6.65, *p*=0.25 | *X*^2^=7.83, *p*=0.17 |
| **CHD lesion present** | **CHD category** | ***X*^2^=5.91, *p*=0.02** | *X*^2^=3.08, *p*=0.08 | ***X*^2^=5.22, *p*=0.02** | *X*^2^=0.01, *p*=0.92 |
|  | **Teralogy of Fallot** | *X*^2^=0.79, *p*=0.37 | *X*^2^=0.05, *p*=0.82 | *X*^2^=2.10, *p*=0.15 | *X*^2^=0.60, *p*=0.44 |
|  | **Double outlet right ventricle** | ***X*^2^=4.18, *p*=0.04** | *X*^2^=0.41, *p*=0.52 | *X*^2^=0.82, *p*=0.36 | *X*^2^=0.58, *p*=0.45 |
|  | **Atrial septal defect- Secundum** | *X*^2^=0.16, *p*=0.69 | *X*^2^=1.37, *p*=0.24 | ***X*^2^=7.87, *p*=0.01** | *X*^2^=0.21, *p*=0.64 |
|  | **Hypoplastic left heart syndrome** | *X*^2^=0.23, *p*=0.63 | *X*^2^=0.46, *p*=0.50 | *X*^2^=0.64, *p*=0.42 | *X*^2^=0.18, *p*=0.67 |
|  | **Coarctation of the aorta** | *X*^2^=0.18, *p*=0.67 | *X*^2^=0.01, *p*=0.93 | *X*^2^=0.18, *p*=0.67 | *X*^2^=3.75, *p*=0.05 |
|  | **D-transposition great arteries** | *X*^2^=0.88, *p*=0.35 | ***X*^2^=8.88, *p*=0.003** | *X*^2^=0.86, *p*=0.35 | *X*^2^=1.85, *p*=0.17 |
|  | **Isolated ventricular septal defect** | *X*^2^=0.57, *p*=0.45 | *X*^2^=1.87, *p*=0.17 | *X*^2^=2.04, *p*=0.15 | *X*^2^=0.60, *p*=0.44 |
|  | **Atrioventricular canal** | *X*^2^=0.18, *p*=0.67 | *X*^2^=0.91, *p*=0.34 | *X*^2^=0.18, *p*=0.67 | *X*^2^=1.20, *p*=0.27 |
|  | **Heterotaxy** | *X*^2^=0.07, *p*=0.80 | *X*^2^=0.58, *p*=0.45 | *X*^2^=0.12, *p*=0.73 | *X*^2^=0.65, *p*=0.42 |
| **LOF**  **variant present** | **HBE** | *X*^2^=1.32, *p*=0.25 | *X*^2^=0.07, *p*=0.80 | ***X*^2^=6.65, *p*=0.01** | *X*^2^=0.55, *p*=0.46 |
|  | **CHR** | *X*^2^=0.00, *p*=0.97 | *X*^2^=1.30, *p*=0.25 | *X*^2^=0.01, *p*=0.94 | *X*^2^=0.01, *p*=0.92 |
|  | **NDD** | *X*^2^=0.13, *p*=0.72 | *X*^2^=0.46, *p*=0.50 | *X*^2^=0.01, *p*=0.94 | ***X*^2^=5.79, *p*=0.02** |
|  | **H-pLI** | *X*^2^=0.26, *p*=0.61 | *X*^2^=1.32, *p*=0.25 | ***X*^2^=4.56, *p*=0.03** | *X*^2^=0.02, *p*=0.88 |
| **Medical**  **history** | **MRI findings** | *X*^2^=1.35, *p*=0.25 | *X*^2^=0.30, *p*=0.58 | *X*^2^=0.04, *p*=0.83 | *X*^2^=2.21, *p*=0.14 |
|  | **# surgeries** | *H*=0.39, *p*=0.53 | *H*=0.48, *p*=0.49 | *H*=2.30, *p*=0.13 | *H*=2.27, *p*=0.13 |
|  | **# open heart surgeries** | *H*=1.51, *p*=0.22 | *H*=0.66, *p*=0.42 | *H*=2.35, *p*=0.12 | *H*=0.51, *p*=0.48 |
|  | **Age of 1^st^ hospital admission** | *H*=2.27, *p*=0.13 | *H*=0.11, *p*=0.74 | *H*=0.79, *p*=0.37 | *H*=0.69, *p*=0.41 |
|  | **Age of 1^st^ open heart surgery** | *H*=1.80, *p*=0.18 | *H*=1.12, *p*=0.29 | *H*=2.00, *p*=0.16 | *H*=0.63, *p*=0.43 |
| **WRAT-4** | **Word reading** | *F*=0.16, *p*=0.69 | *F*=0.84, *p*=0.36 | *F*=0.14, *p*=0.71 | *F*=0.37, *p*=0.54 |
|  | **Sentence comprehension** | *F*=0.88, *p*=0.35 | *F*=1.61, *p*=0.21 | *F*=2.17, *p*=0.15 | *F*=0.42, *p*=0.52 |
|  | **Spelling** | *F*=0.19, *p*=0.67 | *F*=2.08, *p*=0.16 | *F*=0.01, *p*=0.91 | *F*=0.06, *p*=0.81 |
|  | **Math computation** | *F*=0.12, *p*=0.73 | *F*=0.28, *p*=0.60 | *F*=0.01, *p*=0.94 | *F*=0.57, *p*=0.45 |
|  | **Reading composite** | *F*=0.51, *p*=0.48 | *F*=0.07, *p*=0.80 | *F*=0.84, *p*=0.36 | *F*=0.48, *p*=0.49 |
| **Beery-VMI** | **VMI** | *F*=0.91, *p*=0.34 | *F*=0.02, *p*=0.88 | *F*=1.40, *p*=0.24 | *F*=0.57, *p*=0.45 |
| **W-IQ** | **Verbal comprehension index** | *F*=0.55, *p*=0.46 | *F*=0.24, *p*=0.62 | *F*=0.52, *p*=0.47 | *F*=0.23, *p*=0.63 |
|  | **Perceptual/fluid reasoning index** | *F*=0.11, *p*=0.74 | *F*=2.51, *p*=0.12 | *F*=2.18, *p*=0.15 | *F*=0.14, *p*=0.71 |
|  | **Processing speed index** | *F*=0.01, *p*=0.92 | *F*=0.18, *p*=0.67 | *H*=0.01, *p*=0.91 | *F*=0.14, *p*=0.71 |
|  | **Full-scale intelligence quotient** | *F*=0.27, *p*=0.60 | *F*=0.34, *p*=0.56 | *F*=0.38, *p*=0.54 | *F*=0.26, *p*=0.61 |
| **WRAML-2** | **Immediate recall (story memory)** | *H*=3.66, *p*=0.06 | *F*=1.20, *p*=0.28 | *H*=0.94, *p*=0.33 | *H*=2.68, *p*=0.10 |
|  | **Immediate recall (picture memory)** | *F*=2.40, *p*=0.13 | *F*=0.14, *p*=0.71 | *F*=3.61, *p*=0.06 | *H*=0.05, *p*=0.82 |
|  | **Delayed recall (picture memory)** | *H*=1.61, *p*=0.20 | *F*=0.76, *p*=0.39 | *H*=0.56, *p*=0.45 | *F*=0.36, *p*=0.55 |
|  | **Delayed recall (story memory)** | *H*=1.77, *p*=0.18 | *F*=0.22, *p*=0.64 | *H*=0.39, *p*=0.53 | *F*=1.80, *p*=0.19 |
|  | **Delayed recall (story recognition)** | *H*=0.61, *p*=0.43 | *F*=0.01, *p*=0.93 | *H*=0.06, *p*=0.81 | *H*=1.62, *p*=0.20 |
| **D-KEFS** | **Letter fluency** | *H*=0.31, *p*=0.58 | *F*=0.75, *p*=0.39 | *F*=0.08, *p*=0.78 | *F*=1.89, *p*=0.18 |
|  | **Category fluency** | *H*=0.97, *p*=0.32 | *H*=2.24, *p*=0.13 | *F*=0.13, *p*=0.72 | ***F*=4.64, *p*=0.04** |
|  | **Category switching accuracy** | *H*=1.52, *p*=0.22 | *H*=1.17, *p*=0.28 | *H*=0.66, *p*=0.42 | *F*=0.57, *p*=0.45 |
|  | **Visual scanning** | *H*=0.66, *p*=0.42 | *H*=0.19, *p*=0.67 | *H*=0.01, *p*=0.94 | *H*=0.78, *p*=0.38 |
|  | **Number sequencing** | *H*=1.78, *p*=0.18 | *H*=0.13, *p*=0.72 | *H*=0.22, *p*=0.64 | *H*=1.63, *p*=0.20 |
|  | **Letter sequencing** | *H*=0.05, *p*=0.82 | *H*=0.29, *p*=0.59 | *H*=0.08, *p*=0.78 | *H*=1.04, *p*=0.31 |
|  | **Number-letter switching** | *H*=0.44, *p*=0.50 | *H*=0.29, *p*=0.59 | *H*=2.07, *p*=0.15 | *H*=0.04, *p*=0.84 |
|  | **Tower total achievement** | *H*=0.05, *p*=0.82 | *F*=1.51, *p*=0.23 | *H*=3.12, *p*=0.08 | *H*=0.01, *p*=0.91 |
| **WIAT-III** | **Oral language composite** | ***F*=7.28, *p*=0.01** | *F*=0.02, *p*=0.90 | *H*=0.20, *p*=0.65 | *F*=0.25, *p*=0.62 |
| **Vineland-3** | **Socialization** | *F*=0.99, *p*=0.32 | *H*=1.49, *p*=0.22 | ***F*=17.62, *p*=0.0001** | *F*=2.50, *p*=0.12 |
|  | **Communication** | *H*=0.61, *p*=0.43 | *F*=2.84, *p*=0.10 | *H*=0.66, *p*=0.42 | ***F*=5.63, *p*=0.02** |
|  | **Daily living skills** | *F*=0.23, *p*=0.63 | *F*=2.79, *p*=0.10 | *F*=0.71, *p*=0.40 | *F*=2.56, *p*=0.12 |
|  | **Adaptive behaviour composite** | *H*=0.34, *p*=0.56 | *H*=3.21, *p*=0.07 | *F*=1.36, *p*=0.25 | *F*=2.31, *p*=0.14 |

sMRI: structural magnetic resonance imaging; CHD: congenital heart disease; MRI: magnetic resonance imaging; HBE: high brain expressed gene; CHR: chromatin remodelling gene; NDD: newly identified as a dominant neurodevelopmental disability gene or a single variant in a recessive NDD gene; H-pLI: constrained gene with a high probability of intolerance to loss; WRAT-4: Wide Range Achievement Test, Fourth Edition; Beery-Buktenica Developmental Test of Visual-Motor Integration, Sixth Edition; W-IQ: Wechsler intelligence quotient scales; WRAML-2: Wide Range Assessment of Memory and Learning, Second Edition; D-KEFS: Delis Kaplan Executive Function System; WIAT-III: Wechsler Individual Achievement Test’s oral language composite; Vineland-3: Vineland Adaptive Behavior Scales, Third Edition; ^1^1=<$24,999/year, 2=$25,000-$49,999/year, 3=$50,000-$74,999/year, 4=$75,000-$99,999/year, 5=$100,000-$149,999/year, 6=>$150,000/year; ^2^1=high school or under/other, 2=some college/college, 3=post graduate degree

**Supplemental Table 7**: Statistical details examining differences in the sample characteristics, behavioural measures, and medical history measures between each pair of leaves in the dMRI dendrogram. For the continuous measures, one-way ANOVAs (indicated by the *F*-statistic) or Kruskal-Wallis (indicated by the *H*-statistic) were performed depending on normality. Significant (*p*<0.05) effects are bolded.

|  |  | **2-cluster** | **3-cluster** | **4-cluster** | **5-cluster** |
| --- | --- | --- | --- | --- | --- |
| **Demo-**  **graphics** | **Age** | *H*=0.08, *p*=0.78 | *H*=0.32, *p*=0.57 | *F*=0.13, *p*=0.72 | *F*=0.01, *p*=0.93 |
|  | **Sex** | *X*^2^=0.09, *p*=0.76 | *X*^2^=2.37, *p*=0.12 | *X*^2^=0.99, *p*=0.32 | *X*^2^=0.21, *p*=0.64 |
|  | **Household income level^1^** | *W*=0.22, *p*=0.64 | ***W*=4.25, *p*=0.04** | *W*=0.03, *p*=0.88 | *W*=0.26, *p*=0.61 |
|  | **Mother’s education level^2^** | *W*=1.31, *p*=0.25 | *W*=0.34, *p*=0.56 | *W*=0.01, *p*=0.94 | *W*=0.21, *p*=0.65 |
|  | **Tier** | *X*^2^=0.19, *p*=0.66 | *X*^2^=1.13, *p*=0.29 | *X*^2^=1.55, *p*=0.21 | *X*^2^=0.21, *p*=0.64 |
|  | **MRI Site** | *X*^2^=3.78, *p*=0.44 | *X*^2^=2.46, *p*=0.65 | *X*^2^=5.13, *p*=0.27 | *X*^2^=1.48, *p*=0.83 |
| **CHD lesion present** | **CHD category** | *X*^2^=0.06, *p*=0.80 | *X*^2^=0.42, *p*=0.51 | *X*^2^=0.11, *p*=0.74 | *X*^2^=0.29, *p*=0.59 |
|  | **Teralogy of Fallot** | *X*^2^=0.00, *p*=0.99 | *X*^2^=1.52, *p*=0.22 | *X*^2^=0.30, *p*=0.58 | *X*^2^=3.41, *p*=0.06 |
|  | **Double outlet right ventricle** | ***X*^2^=4.68, *p*=0.03** | *X*^2^=0.11, *p*=0.74 | *X*^2^=2.28, *p*=0.13 | *X*^2^=0.56, *p*=0.46 |
|  | **Atrial septal defect- Secundum** | *X*^2^=1.80, *p*=0.18 | *X*^2^=0.01, *p*=0.94 | *X*^2^=0.11, *p*=0.74 | *X*^2^=1.79, *p*=0.18 |
|  | **Hypoplastic left heart syndrome** | *X*^2^=3.34, *p*=0.07 | *X*^2^=0.68, *p*=0.41 | *X*^2^=0.00, *p*=0.96 | *X*^2^=0.00, *p*=0.95 |
|  | **Coarctation of the aorta** | *X*^2^=0.08, *p*=0.78 | *X*^2^=0.10, *p*=0.76 | *X*^2^=0.00, *p*=0.96 | *X*^2^=1.17, *p*=0.28 |
|  | **D-transposition great arteries** | *X*^2^=1.49, *p*=0.22 | *X*^2^=0.24, *p*=0.63 | *X*^2^=0.00, *p*=0.94 | *X*^2^=0.22, *p*=0.64 |
|  | **Isolated ventricular septal defect** | *X*^2^=1.49, *p*=0.22 | *X*^2^=1.52, *p*=0.22 | *X*^2^=0.80, *p*=0.37 | *X*^2^=2.58, *p*=0.11 |
|  | **Atrioventricular canal** | *X*^2^=0.32, *p*=0.57 | *X*^2^=0.02, *p*=0.90 | *X*^2^=0.30, *p*=0.58 | *X*^2^=0.22, *p*=0.64 |
|  | **Heterotaxy** | *X*^2^=0.36, *p*=0.55 | *X*^2^=0.65, *p*=0.42 | *X*^2^=0.17, *p*=0.68 | ***X*^2^=4.11, *p*=0.04** |
| **LOF**  **variant** | **HBE** | ***X*^2^=7.99, *p*=0.005** | *X*^2^=3.46, *p*=0.06 | *X*^2^=0.26, *p*=0.61 | *X*^2^=0.00, *p*=0.95 |
|  | **CHR** | *X*^2^=0.00, *p*=0.94 | *X*^2^=0.02, *p*=0.90 | *X*^2^=0.44, *p*=0.51 | *X*^2^=0.22, *p*=0.64 |
|  | **NDD** | *X*^2^=0.08, *p*=0.78 | *X*^2^=0.10, *p*=0.76 | *X*^2^=0.00, *p*=0.96 | *X*^2^=1.17, *p*=0.28 |
|  | **H-pLI** | *X*^2^=0.02, *p*=0.90 | *X*^2^=3.18, *p*=0.07 | *X*^2^=0.02, *p*=0.90 | *X*^2^=0.18, *p*=0.67 |
| **Medical**  **history** | **MRI findings** | *X*^2^=0.12, *p*=0.73 | *X*^2^=1.35, *p*=0.25 | *X*^2^=0.42, *p*=0.52 | *X*^2^=0.02, *p*=0.88 |
|  | **# surgeries** | *H*=1.34, *p*=0.25 | *H*=0.69, *p*=0.41 | *H*=0.42, *p*=0.52 | *H*=0.18, *p*=0.68 |
|  | **# open heart surgeries** | *H*=2.92, *p*=0.09 | *H*=0.67, *p*=0.41 | *H*=0.73, *p*=0.39 | *H*=3.67, *p*=0.06 |
|  | **Age of 1^st^ hospital admission** | *H*=0.16, *p*=0.69 | *H*=0.01, *p*=0.91 | *H*=0.27, *p*=0.60 | ***H*=4.69, *p*=0.03** |
|  | **Age of 1^st^ open heart surgery** | *H*=0.01, *p*=0.94 | *H*=0.30, *p*=0.58 | *H*=0.43, *p*=0.51 | ***H*=7.09, *p*=0.01** |
| **WRAT-4** | **Word reading** | *F*=0.59, *p*=0.44 | *F*=0.54, *p*=0.47 | *F*=0.15, *p*=0.70 | ***F*=12.51, *p*=0.002** |
|  | **Sentence comprehension** | *F*=0.76, *p*=0.39 | *F*=0.39, *p*=0.54 | *F*=2.64, *p*=0.12 | ***F*=7.14, *p*=0.01** |
|  | **Spelling** | *F*=0.13, *p*=0.72 | *F*=1.22, *p*=0.27 | *F*=2.19, *p*=0.15 | *F*=3.08, *p*=0.09 |
|  | **Math computation** | *F*=1.21, *p*=0.27 | *F*=3.30, *p*=0.08 | *F*=0.14, *p*=0.72 | ***F*=6.54, *p*=0.02** |
|  | **Reading composite** | *F*=0.85, *p*=0.36 | *F*=0.56, *p*=0.46 | *F*=1.44, *p*=0.24 | ***F*=11.60, *p*=0.003** |
| **Beery-VMI** | **VMI** | ***F*=4.26, *p*=0.04** | *F*=0.13, *p*=0.72 | *F*=0.02, *p*=0.89 | ***F*=4.70, *p*=0.04** |
| **W-IQ** | **Verbal comprehension index** | *F*=0.41, *p*=0.52 | *F*=0.35, *p*=0.56 | *F*=0.01, *p*=0.90 | ***F*=4.92, *p*=0.04** |
|  | **Perceptual/fluid reasoning index** | *F*=0.00, *p*=0.99 | *F*=1.10, *p*=0.30 | *F*=0.09, *p*=0.77 | *F*=0.00, *p*=1.00 |
|  | **Processing speed index** | *F*=1.61, *p*=0.21 | *F*=0.27, *p*=0.61 | ***F*=4.82, *p*=0.04** | *F*=2.44, *p*=0.13 |
|  | **Full-scale intelligence quotient** | *F*=0.11, *p*=0.74 | *F*=0.90, *p*=0.35 | *F*=0.22, *p*=0.64 | *F*=4.31, *p*=0.05 |
| **WRAML-2** | **Immediate recall (story memory)** | *H*=0.70, *p*=0.40 | *F*=0.33, *p*=0.57 | *F*=0.89, *p*=0.35 | *F*=0.76, *p*=0.39 |
|  | **Immediate recall (picture memory)** | *F*=0.33, *p*=0.57 | *F*=0.54, *p*=0.47 | *F*=0.01, *p*=0.93 | *F*=0.00, *p*=0.96 |
|  | **Delayed recall (picture memory)** | *H*=0.23, *p*=0.63 | *F*=2.01, *p*=0.16 | *F*=0.00, *p*=0.97 | *H*=0.77, *p*=0.38 |
|  | **Delayed recall (story memory)** | *H*=0.76, *p*=0.38 | *F*=0.14, *p*=0.71 | *H*=0.68, *p*=0.41 | *F*=1.18, *p*=0.29 |
|  | **Delayed recall (story recognition)** | *H*=2.47, *p*=0.12 | *F*=0.13, *p*=0.73 | *F*=0.14, *p*=0.71 | *F*=0.05, *p*=0.82 |
| **D-KEFS** | **Letter fluency** | *H*=3.33, *p*=0.07 | *F*=0.02, *p*=0.90 | ***F*=5.45, *p*=0.03** | *F*=2.19, *p*=0.15 |
|  | **Category fluency** | *H*=2.55, *p*=0.11 | *H*=0.11, *p*=0.74 | ***F*=7.26, *p*=0.01** | ***F*=4.71, *p*=0.04** |
|  | **Category switching accuracy** | *H*=1.26, *p*=0.26 | *H*=0.01, *p*=0.94 | *F*=1.23, *p*=0.28 | ***F*=5.22, *p*=0.03** |
|  | **Visual scanning** | *H*=0.07, *p*=0.79 | *H*=0.08, *p*=0.77 | *F*=0.49, *p*=0.49 | *H*=2.79, *p*=0.09 |
|  | **Number sequencing** | *H*=0.34, *p*=0.56 | *H*=0.39, *p*=0.53 | *H*=0.31, *p*=0.58 | ***H*=5.24, *p*=0.02** |
|  | **Letter sequencing** | *H*=3.44, *p*=0.06 | *H*=0.86, *p*=0.35 | *H*=0.43, *p*=0.51 | ***F*=8.26, *p*=0.01** |
|  | **Number-letter switching** | *H*=0.16, *p*=0.69 | *H*=0.73, *p*=0.39 | *H*=0.17, *p*=0.68 | *H*=1.64, *p*=0.20 |
|  | **Tower total achievement** | *H*=0.00, *p*=0.96 | *H*=0.20, *p*=0.65 | *H*=0.84, *p*=0.36 | *F*=0.00, *p*=0.97 |
| **WIAT-III** | **Oral language composite** | *F*=0.01, *p*=0.94 | *F*=2.36, *p*=0.13 | *F*=0.17, *p*=0.68 | *F*=3.73, *p*=0.07 |
| **Vineland-3** | **Socialization** | *F*=0.85, *p*=0.36 | *F*=0.04, *p*=0.83 | *F*=0.04, *p*=0.85 | *F*=2.65, *p*=0.12 |
|  | **Communication** | *H*=0.09, *p*=0.77 | *H*=0.47, *p*=0.49 | *H*=0.04, *p*=0.83 | *F*=0.12, *p*=0.74 |
|  | **Daily living skills** | *F*=0.00, *p*=0.97 | *F*=1.12, *p*=0.30 | *F*=1.36, *p*=0.25 | *F*=0.31, *p*=0.59 |
|  | **Adaptive behaviour composite** | *H*=0.08, *p*=0.77 | *H*=0.12, *p*=0.73 | *F*=0.93, *p*=0.34 | *H*=0.10, *p*=0.75 |

dMRI: diffusion magnetic resonance imaging; CHD: congenital heart disease; MRI: magnetic resonance imaging; HBE: high brain expressed gene; CHR: chromatin remodelling gene; NDD: newly identified as a dominant neurodevelopmental disability gene or a single variant in a recessive NDD gene; H-pLI: constrained gene with a high probability of intolerance to loss; WRAT-4: Wide Range Achievement Test, Fourth Edition; Beery-Buktenica Developmental Test of Visual-Motor Integration, Sixth Edition; W-IQ: Wechsler intelligence quotient scales; WRAML-2: Wide Range Assessment of Memory and Learning, Second Edition; D-KEFS: Delis Kaplan Executive Function System; WIAT-III: Wechsler Individual Achievement Test’s oral language composite; Vineland-3: Vineland Adaptive Behavior Scales, Third Edition; ^1^1=<$24,999/year, 2=$25,000-$49,999/year, 3=$50,000-$74,999/year, 4=$75,000-$99,999/year, 5=$100,000-$149,999/year, 6=>$150,000/year; ^2^1=high school or under/other, 2=some college/college, 3=post graduate degree

**References**

Avants, B.B., Epstein, C.L., Grossman, M., Gee, J.C., 2008. Symmetric diffeomorphic image registration with cross-correlation: Evaluating automated labeling of elderly and neurodegenerative brain. Med Image Anal 12, 26–41. https://doi.org/10.1016/j.media.2007.06.004

Calinski, T., Harabasz, J., 1974. A Dendrite Method for Cluster Analysis. Commun Stat Simul Comput 3, 1–27. https://doi.org/10.1080/03610917408548446

Chikumbo, O., Granville, V., 2019. Optimal Clustering and Cluster Identity in Understanding High-Dimensional Data Spaces with Tightly Distributed Points. Machine Learning and Knowledge Extraction 2019, Vol. 1, Pages 715-744 1, 715–744. https://doi.org/10.3390/MAKE1020042

Cieslak, M., Cook, P.A., He, X., Yeh, F.C., Dhollander, T., Adebimpe, A., Aguirre, G.K., Bassett, D.S., Betzel, R.F., Bourque, J., Cabral, L.M., Davatzikos, C., Detre, J.A., Earl, E., Elliott, M.A., Fadnavis, S., Fair, D.A., Foran, W., Fotiadis, P., Garyfallidis, E., Giesbrecht, B., Gur, R.C., Gur, R.E., Kelz, M.B., Keshavan, A., Larsen, B.S., Luna, B., Mackey, A.P., Milham, M.P., Oathes, D.J., Perrone, A., Pines, A.R., Roalf, D.R., Richie-Halford, A., Rokem, A., Sydnor, V.J., Tapera, T.M., Tooley, U.A., Vettel, J.M., Yeatman, J.D., Grafton, S.T., Satterthwaite, T.D., 2021. QSIPrep: an integrative platform for preprocessing and reconstructing diffusion MRI data. Nature Methods 2021 18:7 18, 775–778. https://doi.org/10.1038/s41592-021-01185-5

Cox, R.W., Hyde, J.S., 1997. Software tools for analysis and visualization of fMRI data. NMR Biomed 10, 171–178.

Desikan, R.S., Se, F., Fischl, B., Quinn, B.T., Dickerson, B.C., Blacker, D., Buckner, R.L., Dale, A.M., Maguire, R.P., Hyman, B.T., Albert, M.S., Killiany, R.J., 2006. An automated labeling system for subdividing the human cerebral cortex on MRI scans into gyral based regions of interest. Neuroimage 31, 968–980. https://doi.org/10.1016/j.neuroimage.2006.01.021

Dhollander, T., Mito, R., Raffelt, D., Connelly, A., 2019. Improved white matter response function estimation for 3-tissue constrained spherical deconvolution. Proc. Intl. Soc. Mag. Reson. Med 555.

Dhollander, T., Raffelt, D., Connelly, A., 2016. Unsupervised 3-tissue response function estimation from single-shell or multi-shell diffusion MR data without a co-registered T1 image. ISMRM Workshop on Breaking the Barriers of Diffusion MRI 5.

Fischl, B., 2012. FreeSurfer. Neuroimage 62, 774–781. https://doi.org/10.1016/j.neuroimage.2012.01.021

Fischl, B., Salat, D. ~H., Busa, E., Albert, M., Dieterich, M., Haselgrove, C., van der Kouwe, A., Killiany, R., Kennedy, D., Klaveness, S., Montillo, A., Makris, N., Rosen, B., Dale, A. ~M., 2002. Whole brain segmentation: automated labeling of neuroanatomical structures in the human brain. Neuron 33, 341–355.

Fonov, V.S., Evans, A.C., McKinstry, R.C., Almli, C.R., Collins, D.L., 2009. Unbiased nonlinear average age-appropriate brain templates from birth to adulthood. Neuroimage 47, S102. https://doi.org/10.1016/s1053-8119(09)70884-5

Hagler, D.J., Hatton, S.N., Cornejo, M.D., Makowski, C., Fair, D.A., Dick, A.S., Sutherland, M.T., Casey, B.J., Barch, D.M., Harms, M.P., Watts, R., Bjork, J.M., Garavan, H.P., Hilmer, L., Pung, C.J., Sicat, C.S., Kuperman, J., Bartsch, H., Xue, F., Heitzeg, M.M., Laird, A.R., Trinh, T.T., Gonzalez, R., Tapert, S.F., Riedel, M.C., Squeglia, L.M., Hyde, L.W., Rosenberg, M.D., Earl, E.A., Howlett, K.D., Baker, F.C., Soules, M., Diaz, J., de Leon, O.R., Thompson, W.K., Neale, M.C., Herting, M., Sowell, E.R., Alvarez, R.P., Hawes, S.W., Sanchez, M., Bodurka, J., Breslin, F.J., Morris, A.S., Paulus, M.P., Simmons, W.K., Polimeni, J.R., van der Kouwe, A., Nencka, A.S., Gray, K.M., Pierpaoli, C., Matochik, J.A., Noronha, A., Aklin, W.M., Conway, K., Glantz, M., Hoffman, E., Little, R., Lopez, M., Pariyadath, V., Weiss, S.R., Wolff-Hughes, D.L., DelCarmen-Wiggins, R., Feldstein Ewing, S.W., Miranda-Dominguez, O., Nagel, B.J., Perrone, A.J., Sturgeon, D.T., Goldstone, A., Pfefferbaum, A., Pohl, K.M., Prouty, D., Uban, K., Bookheimer, S.Y., Dapretto, M., Galvan, A., Bagot, K., Giedd, J., Infante, M.A., Jacobus, J., Patrick, K., Shilling, P.D., Desikan, R., Li, Y., Sugrue, L., Banich, M.T., Friedman, N., Hewitt, J.K., Hopfer, C., Sakai, J., Tanabe, J., Cottler, L.B., Nixon, S.J., Chang, L., Cloak, C., Ernst, T., Reeves, G., Kennedy, D.N., Heeringa, S., Peltier, S., Schulenberg, J., Sripada, C., Zucker, R.A., Iacono, W.G., Luciana, M., Calabro, F.J., Clark, D.B., Lewis, D.A., Luna, B., Schirda, C., Brima, T., Foxe, J.J., Freedman, E.G., Mruzek, D.W., Mason, M.J., Huber, R., McGlade, E., Prescot, A., Renshaw, P.F., Yurgelun-Todd, D.A., Allgaier, N.A., Dumas, J.A., Ivanova, M., Potter, A., Florsheim, P., Larson, C., Lisdahl, K., Charness, M.E., Fuemmeler, B., Hettema, J.M., Maes, H.H., Steinberg, J., Anokhin, A.P., Glaser, P., Heath, A.C., Madden, P.A., Baskin-Sommers, A., Constable, R.T., Grant, S.J., Dowling, G.J., Brown, S.A., Jernigan, T.L., Dale, A.M., 2019. Image processing and analysis methods for the Adolescent Brain Cognitive Development Study. Neuroimage 202, 116091. https://doi.org/10.1016/J.NEUROIMAGE.2019.116091

Jovicich, J., Czanner, S., Greve, D., Haley, E., van der Kouwe, A., Gollub, R., Kennedy, D., Schmitt, F., Brown, G., MacFall, J., Fischl, B., Dale, A., 2006. Reliability in multi-site structural MRI studies: Effects of gradient non-linearity correction on phantom and human data. Neuroimage 30, 436–443. https://doi.org/DOI: 10.1016/j.neuroimage.2005.09.046

Kellner, E., Dhital, B., Kiselev, V.G., Reisert, M., 2016. Gibbs-ringing artifact removal based on local subvoxel-shifts. Magn Reson Med 76, 1574–1581. https://doi.org/10.1002/mrm.26054

Merlet, S.L., Deriche, R., 2013. Continuous diffusion signal, EAP and ODF estimation via Compressive Sensing in diffusion MRI. Med Image Anal 17, 556–572. https://doi.org/10.1016/J.MEDIA.2013.02.010

Raffelt, D., Dhollander, T., Tournier, J.D., Tabbara, R., Smith, R.E., Pierre, E., Connelly, A., 2017. Bias field correction and intensity normalisation for quantitative analysis of apparent fiber density. Proc Intl Soc Mag Reson Med 25, 3541.

Smith, R.E., Tournier, J.D., Calamante, F., Connelly, A., 2015. SIFT2: Enabling dense quantitative assessment of brain white matter connectivity using streamlines tractography. Neuroimage 119, 338–351. https://doi.org/10.1016/j.neuroimage.2015.06.092

Smith, R.E., Tournier, J.D., Calamante, F., Connelly, A., 2012. Anatomically-constrained tractography: Improved diffusion MRI streamlines tractography through effective use of anatomical information. Neuroimage 62, 1924–1938. https://doi.org/10.1016/j.neuroimage.2012.06.005

Tournier, J.D., Calamante, F., Connelly, A., 2010. Improved probabilistic streamlines tractography by 2nd order integration over fibre orientation distributions. Ismrm 88, 2010.

Tournier, J.D., Calamante, F., Gadian, D.G., Connelly, A., 2004. Direct estimation of the fiber orientation density function from diffusion-weighted MRI data using spherical deconvolution. Neuroimage 23, 1176–1185. https://doi.org/10.1016/j.neuroimage.2004.07.037

Tournier, J.D., Yeh, C.H., Calamante, F., Cho, K.H., Connelly, A., Lin, C.P., 2008. Resolving crossing fibres using constrained spherical deconvolution: Validation using diffusion-weighted imaging phantom data. Neuroimage 42, 617–625. https://doi.org/10.1016/j.neuroimage.2008.05.002

Tustison, N.J., Avants, B.B., Cook, P.A., Zheng, Y., Egan, A., Yushkevich, P.A., Gee, J.C., 2010. N4ITK: Improved N3 bias correction. IEEE Trans Med Imaging 29, 1310–1320. https://doi.org/10.1109/TMI.2010.2046908

Veraart, J., Novikov, D.S., Christiaens, D., Ades-aron, B., Sijbers, J., Fieremans, E., 2016. Denoising of diffusion MRI using random matrix theory. Neuroimage 142, 394–406. https://doi.org/10.1016/j.neuroimage.2016.08.016

Wald, L., Schmitt, F., Dale, A., 2001. Systematic spatial distortion in MRI due to gradient non-linearities. Neuroimage 6 Supplement, 50. https://doi.org/10.1016/S1053-8119(01)91393-X

Zhang, Y., Brady, M., Smith, S., 2001. Segmentation of brain MR images through a hidden Markov random field model and the expectation-maximization algorithm. IEEE Trans Med Imaging 20, 45–57. https://doi.org/10.1109/42.906424
